# Systematic analysis of some Astereae (Asteraceae) species by Integrating pollen morphology and molecular evidence

**DOI:** 10.3389/fpls.2025.1558995

**Published:** 2025-04-08

**Authors:** Tianmeng Qu, Gan Xie, Xinyi Zheng, Xinyu Chen, Yanru Zhang, Lili Lu, Zhixi Fu

**Affiliations:** ^1^ Key Laboratory of Land Resources Evaluation and Monitoring in Southwest, Sichuan Normal University, Ministry of Education, Chengdu, China; ^2^ College of Life Sciences, Sichuan Normal University, Chengdu, China; ^3^ Big Data and AI Research Center of Biodiversity Conservation, Institute of Botany, Chinese Academy of Sciences, Beijing, China; ^4^ Key Laboratory of Vertebrate Evolution and Human Origins, Institute of Vertebrate Paleontology and Paleoanthropology, Chinese Academy of Sciences, Beijing, China; ^5^ Sustainable Development Research Center of Resources and Environment of Western Sichuan, Sichuan Normal University, Chengdu, China

**Keywords:** taxonomy, *Aster*, *Erigeron*, SEM, phylogeny, cluster analysis

## Abstract

Astereae, the second-largest tribe within Asteraceae, includes numerous species of economic and medicinal importance. While comprehensive systematic studies have been conducted on Astereae classification, certain controversies remain unresolved. The taxonomic boundaries between alpine *Aster* and *Erigeron* are uncertain due to their morphological similarity, and the systematic placement of *Formania mekongensis* remains debated. To address these issues, we applied a combination of morphological and molecular phylogenetic approaches. 21 species from 12 genera within Astereae were selected based on a morphological and molecular phylogenetic framework. Sampling, experiments, photography, and measurements were conducted using standardized methods, resulting in 12 pollen trait parameters. These parameters were then used to construct a hierarchical dendrogram of pollen morphology. A molecular phylogeny was constructed based on ITS sequences to further elucidate the systematic relationships among these species. The results revealed that pollen morphology provides valuable insights into subtribal classifications. Significant differences in pollen size and spine morphology were observed between *Aster* and *Erigeron*, with the former exhibiting larger pollen grains with long, broad, and sparsely distributed spines. Clustering results also provided the first palynological evidence for placing *F. mekongensis* within Asterinae. This study underscores the importance of integrating pollen morphology and molecular evidence to refine the classification and phylogeny of Astereae.

## 1 Introduction

Morphological and molecular data provide essential evidence for estimating evolutionary relationships in plants (Sattler and Rutishauser, 1997, 2022; Janaćković et al., 2019; Bog et al., 2020; Sattler, 2022; Zhang et al., 2024). While molecular techniques offer insights at the genetic level, morphological analysis remains fundamental for understanding phenotypic evolution (Wanninger, 2015). The combined use of these approaches has improved the resolution of classification and phylogenetic relationships (Wortley and Scotland, 2006; Huang et al., 2013; Bapst et al., 2018; Keating et al., 2023). Pollen morphology serves as a valuable tool for species identification and classification due to its conserved characteristics (Wang and Wang, 1983; Dajoz et al., 1991; Blackmore, 2007; Lin et al., 2023). These stable features make pollen morphology particularly useful in plant systematics (Hesse and Blackmore, 2013; Lacourse et al., 2016; Bahadur et al., 2018). Integrating morphological data with other approaches may further advance our understanding in this field.

Astereae is the second-largest tribe within Asteraceae, comprising approximately 222 genera and 3,100 species (Ling et al., 1985; Anderberg et al., 2007; Funk et al., 2009). Its classification remains challenging, partly due to the limited sampling (Brouillet et al., 2009; Li et al., 2012). The taxonomic framework of Astereae has undergone notable revisions since Bentham’s (1873) initial division into six subtribes: Solidagininae, Grangeinae, Bellidinae, Asterinae, Conyzinae, and Baccharidinae. Zhang and Bremer (1993) later redefined this classification, recognizing Grangeinae as basal and consolidating the remaining taxa into two primary subtribes: Solidagininae and Asterinae. Nesom (1994) further refined the classification system by establishing 14 subtribes based on morphological features. Despite these advances, several taxonomic uncertainties persist. Notably, the morphological convergence between alpine *Aster* and *Erigeron* has resulted in ambiguous generic boundaries (Cronquist, 1955; Nesom, 1994). The systematic position of the monophyletic *F. mekongensis* has puzzled taxonomists for a long time. While Shi and Fu (1983) classified it within Chrysantheminae (Anthemideae), Chen and Brouillet (2011b) regarded its taxonomic placement as unresolved. Molecular phylogenetic evidence later prompted Fu et al. (2016) to suggest its inclusion in Astereae, and more recently, Nesom (2020) assigned it to the newly established subtribe Formaniinae. These studies highlight the necessity for integrated systematic approaches in Astereae classification.

Pollen morphology has long been employed to address taxonomic questions within the Asteraceae (Tellería, 2017; Younis et al., 2021; Lu et al., 2022; Usma et al., 2022; Ali et al., 2023; Hayat et al., 2023). Key pollen characteristics, including size, shape, aperture type, and exine ornamentation, provide valuable insights for taxonomic classification (Ahmad et al., 2018; Reshmi and Rajalakshm, 2019). Wortley et al. (2007) demonstrated its importance in resolving the classification of problematic taxa. Peng et al. (2023) used pollen morphology to examine *Blumea* and *Cyathocline*, revealing discrepancies between palynological evidence and molecular phylogenetic analyses in certain groups. Nevertheless, research on the pollen morphology of Astereae remains limited (Zhang and Zhou, 2016). Few studies have explored the integration of pollen data with molecular evidence, and such combined approaches may help clarify taxonomic boundaries within the tribe.

This study aims to explore the role of pollen traits in the classification of Astereae by integrating pollen morphological data with molecular phylogenetic frameworks. Systematic sampling was conducted on 12 pollen traits across 21 representative species. The specific objectives are as follows: (1) to compare the clustering dendrogram of pollen traits with the molecular phylogenetic tree and evaluate the relevance of pollen traits in systematics; (2) to analyze the pollen morphological differences and phylogenetic relationships among subtribes and genera within Astereae; and (3) to provide foundational pollen data for the taxonomic study of Astereae. This work presents a new perspective on Astereae classification and contributes to the integration of morphological and molecular evidence.

## 2 Materials and methods

### 2.1 Sampling strategy

To systematically analyze pollen morphological variation in Astereae, we conducted light microscopy (LM) and scanning electron microscopy (SEM) examinations, following the phylogenetic frameworks for Asteraceae outlined by Li et al. (2012). The subtribal classification was based on Anderberg et al. (2007), which employed morphological diagnostic characters for systematic identification. A total of 21 taxonomically representative species spanning *Aster*, *Erigeron*, and related genera, were included in this study. Specimens were selected from voucher sheets in the PE herbarium at the Institute of Botany, Chinese Academy of Sciences (**Table 1**). All pollen samples, along with the scientific names of genera and species, were verified against the *Flora of China* (Shi et al., 2011) and *Plants of the World Online* (POWO, https://powo.science.kew.org/, last access: 1 March 2025).

**Table 1:** List of the voucher specimens in PE Herbarium, Institute of Botany, Chinese Academy of Sciences and the GenBank Numbers.

Subtribes	Genera	Species	Collection Site	Collection Date	Collector	Specimen barcodes	GenBank accession number (ITS)
Asterinae	*Arctogeron* DC.	*Arctogeron gramineum* (L.) DC.	Nei Mongol, China	2010.06.09	G. M. Zhou	PE 01885469	JN315928
Asterinae	*Callistephus* Cass.	*Callistephus chinensis* (L.) Nees	Beijing, China	2004.08.18	L. Q. Li et al.	PE 01776740	KP175224
Conyzinae	*Eschenbachia* Moench	*Eschenbachia japonica* (Thunb.) J. Kost.	Chongqing, China	1957.04.26	G. F. Li	PE 00300678	JN315938
Grangeinae	*Dichrocephala* L’Hér. ex DC.	*Dichrocephala benthamii* C. B. Clarke	Guizhou, China	1986.07.06	Beijing Youth Team	PE 01822413	MH808122
	*Formania* W. W. Sm. & J. Small	*Formania mekongensis* W. W. Sm. & J. Small	Sichuan, China	1981.08.29	Qinghai-Tibet Team	PE 01190762	AY572951
Asterinae	*Galatella* Cass.	*Galatella angustissima* (Tausch) Novopokr.	Xinjiang, China	1956.08.03	Xinjiang Team	PE 01824422	KJ711880
Lagenophorinae	*Myriactis* Less.	*Myriactis wallichii* Less.	Yunnan, China	1940.10	R. C. Qin	PE 00301584	LC027399
	*Nannoglottis* Maxim.	*Nannoglottis carpesioides* Maxim.	Shaanxi, China	1955.06.28	Taibai Team	PE 01648838	AY017161
Solidagininae	*Solidago* L.	*Solidago altissima* L.	USA	2005.09.14	L. R. Phillippe	PE 01505163	JN204176
Asterinae	*Turczaninovia* DC.	*Turczaninovia fastigiata* (Fisch.) DC.	Jilin, China	1960.08.27	J. X. Ye	PE 01822716	JN543739
Asterinae	*Aster* L.	*Aster ageratoides* Turcz.	Hebei, China	1935.08	Y. Liu	PE 00247756	ON427115
		*Aster yunnanensis* Franch.	Xizang, China	1990.07.23	J. S. Yang	PE 01822320	JN543853
		*Aster brachytrichus* Franch.	Sichuan, China	2011.07.21	Y. S. Chen & Y. C. Bi	PE 02016463	JN543838
		*Aster taliangshanensis* Y. Ling	Sichuan, China	1975.08.19		PE 01831078	JN543772
		*Aster turbinatus* S. Moore	Fujian, China	1987.08.31	L. G. Lin	PE 01822347	JN543814
		*Aster homochlamydeus* Hand.-Mazz.	Sichuan, China	1951.09.07	W. G. Hu & Z. He	PE 01825938	JN543784
		*Aster altaicus* Willd.	China	1956.09.03	Yellow River Investigation Team	PE 01607346	MT922723
Conyzinae	*Erigeron* L.	*Erigeron lonchophyllus* Hook.	USA	2011.07.31	R. R. Halse	PE 01920570	AF118505
		*Erigeron strigosus* Muhl. ex Willd.	USA	2015.07.14	R. R. Halse	PE 02110955	AF118490
		*Erigeron acris* L.	Canada	1977.08.05	J. M. Gillett & M. Boudreau	PE 00246145	ON527430
		*Erigeron acris* subsp. *politus* (Fr.) H. Lindb.	Xinjiang, China	2007.07.18	S. V. Smirnov et al.	PE 02016791	KJ711906

Due to the absence of ITS data for *Solidago altissima* L. in the NCBI database, *Solidago decurrens* L. was used as a reference species instead.

### 2.2 Collection of pollen morphological trait data

**Figure 1:**
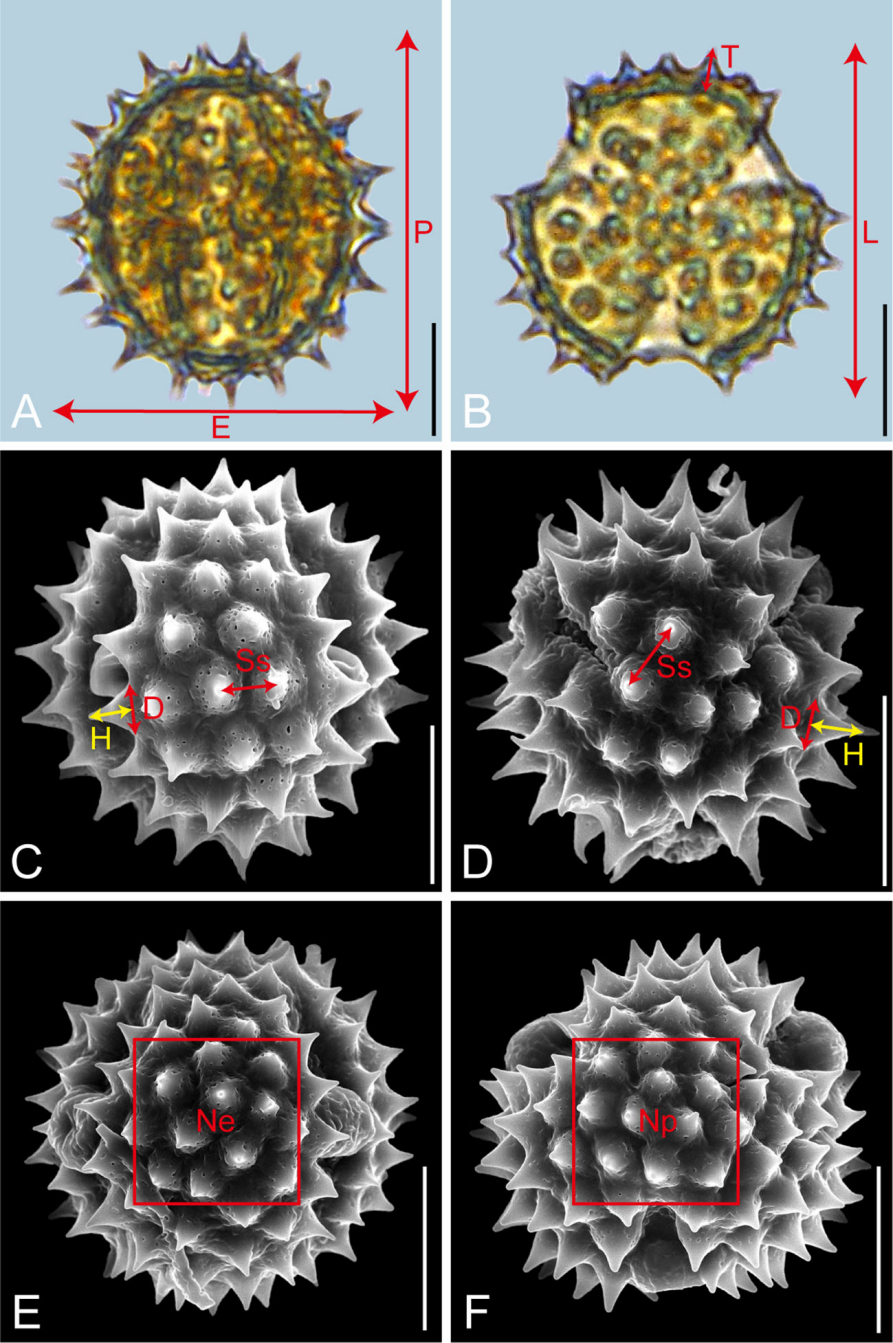
Graphical illustration of measured pollen morphological traits in Astereae (**A**, *Myriactis wallichii*; **B**, *Erigeron lonchophyllus*; **C**, *Galatella angustissima*; **D**, *Arctogeron gramineum*; **E**, **F**, *Aster altaicus*). Scale bar in LM and SEM overview 10µm, and in SEM close-up 1µm.

Pollen samples were acetolysed by the standard methods (Erdtman, 1960) and fixed in glycerine jelly. Processing and observation under LM and SEM followed standard procedures (Wang et al., 1995). The pollen grains were observed and photographed at a magnification of ×600 under LM (Leica DM 4000) and at an accelerating voltage of 30 kV under SEM (Hitachi S-4800). Descriptions of pollen morphology were based on the terminology systems proposed by Halbritter et al. (2018) and Hesse et al. (2009). As shown in **Figure 1**, the pollen morphological traits measured under LM included P: polar length in equatorial view; E: equatorial width in equatorial view; P/E; T: exine thickness in polar view; L: pollen length in polar view; T/L. Each trait was measured on 20 pollen grains per species. The exine ornamentation traits measured under SEM included D: diameter of spinule base; H: spinule height; D/H; Ss: spinule spacing. For these four traits, measurements were taken on five pollen grains per trait, with four randomly selected regions per pollen grain, yielding 20 measurements per trait (Lu et al., 2022). Given the sample sizes of *Galatella angustissima* (n=16) and *Aster taliangshanensis* (n=15), the mean values of the available data were used to supplement the missing samples, ensuring a complete and representative sample size of 20 for statistical analysis. The sexine/nexine (S/N) ratio was measured based on LM observations of the exine structure (**Table 2**; **Supplementary Data**).

**Table 2:** Quantitative morphological traits of pollen in 21 selected species.

Species	Np	Ne	Ss (μm)	D (μm)	H (μm)	D/H	T (μm)	L (μm)	T/L	P (μm)	E (μm)	P/E	S/N	Perforations at base
*Arctogeron gramineum* (L.) DC.	10	12	3.96 ± 0.66	2.83 ± 0.25	3.41 ± 0.27	0.83 ± 0.07	5.00 ± 0.53	30.73 ± 1.54	0.16 ± 0.02	30.11 ± 1.50	26.93 ± 1.27	1.12 ± 0.05	2	1-2
*Callistephus chinensis* (L.) Nees	9	10	4.43 ± 0.72	3.09 ± 0.33	3.68 ± 0.52	0.85 ± 0.08	4.57 ± 0.41	34.74 ± 1.45	0.13 ± 0.01	33.94 ± 1.82	32.45 ± 1.39	1.05 ± 0.06	1.5	2-3
*Eschenbachia japonica* (Thunb.) J. Kost.	19	21	3.05 ± 0.49	2.34 ± 0.21	2.45 ± 0.35	0.97 ± 0.14	3.58 ± 0.35	25.80 ± 1.19	0.14 ± 0.01	23.18 ± 1.04	22.19 ± 0.92	1.05 ± 0.04	1.5	1-2
*Dichrocephala benthamii* C. B. Clarke	15	9	3.37 ± 0.92	2.61 ± 0.44	2.83 ± 0.55	0.93 ± 0.08	3.90 ± 0.37	23.90 ± 1.62	0.16 ± 0.01	22.86 ± 2.52	21.93 ± 2.08	1.04 ± 0.08	2	1-2
*Formania mekongensis* W.W.Sm. & J.Small	9	9	4.05 ± 0.41	2.83 ± 0.26	2.78 ± 0.23	1.02 ± 0.09	4.64 ± 0.44	28.22 ± 1.73	0.16 ± 0.01	26.05 ± 1.43	25.26 ± 2.30	1.04 ± 0.11	2.5	1
*Galatella angustissima* (Tausch) Novopokr.	9	8	4.29 ± 0.52	3.12 ± 0.32	3.22 ± 0.27	0.97 ± 0.09	5.01 ± 0.49	34.71 ± 2.60	0.14 ± 0.01	34.17 ± 1.25	31.37 ± 1.77	1.09 ± 0.05	2	2
*Myriactis wallichii* Less.	10	10	4.16 ± 0.62	2.97 ± 0.35	3.74 ± 0.52	0.80 ± 0.07	5.50 ± 0.54	32.33 ± 2.16	0.17 ± 0.01	32.06 ± 2.07	30.32 ± 1.75	1.06 ± 0.05	2.5	1
*Nannoglottis carpesioides* Maxim.	9	8	4.61 ± 0.74	3.03 ± 0.31	3.66 ± 0.34	0.83 ± 0.06	5.51 ± 0.46	33.33 ± 1.23	0.17 ± 0.01	32.12 ± 2.04	30.07 ± 1.93	1.07 ± 0.07	2	0-1
*Solidago altissima* L.	15	14	3.67 ± 0.69	2.67 ± 0.23	2.88 ± 0.18	0.93 ± 0.09	4.28 ± 0.32	26.76 ± 1.24	0.16 ± 0.01	25.70 ± 2.09	25.38 ± 1.08	1.01 ± 0.05	2.5	1-2
*Turczaninovia fastigiata* (Fisch.) DC.	10	9	4.24 ± 0.45	3.11 ± 0.27	3.41 ± 0.38	0.92 ± 0.12	4.99 ± 0.51	28.34 ± 1.05	0.18 ± 0.01	26.04 ± 1.12	28.25 ± 1.28	0.92 ± 0.02	2.5	2
*Aster ageratoides* Turcz.	10	9	3.87 ± 0.40	2.65 ± 0.17	2.98 ± 0.21	0.89 ± 0.05	4.62 ± 0.47	32.59 ± 1.51	0.14 ± 0.01	31.35 ± 1.35	29.43 ± 1.88	1.07 ± 0.07	1.5	0-1
*Aster yunnanensis* Franch.	10	9	4.15 ± 0.61	3.08 ± 0.36	3.28 ± 0.25	0.94 ± 0.09	4.64 ± 0.34	28.22 ± 1.28	0.16 ± 0.01	26.19 ± 1.04	28.24 ± 1.58	0.93 ± 0.06	2	2
*Aster brachytrichus* Franch.	10	9	4.20 ± 0.83	3.14 ± 0.24	3.11 ± 0.37	1.02 ± 0.10	4.45 ± 0.39	29.43 ± 1.61	0.15 ± 0.01	27.17 ± 1.65	28.01 ± 1.73	0.97 ± 0.07	2	0-2
*Aster taliangshanensis* Y. Ling	8	8	5.16 ± 0.66	3.48 ± 0.39	4.14 ± 0.44	0.84 ± 0.07	6.36 ± 0.63	39.72 ± 2.24	0.16 ± 0.01	35.69 ± 2.32	35.32 ± 1.96	1.01 ± 0.09	2.5	2
*Aster turbinatus* S. Moore	8	10	3.83 ± 0.56	2.86 ± 0.34	3.28 ± 0.42	0.88 ± 0.08	5.04 ± 0.55	32.17 ± 2.58	0.16 ± 0.01	28.66 ± 2.03	29.88 ± 1.52	0.96 ± 0.08	2.5	1-2
*Aster homochlamydeus* Hand.-Mazz.	8	9	4.44 ± 0.76	2.98 ± 0.30	3.72 ± 0.39	0.80 ± 0.06	5.81 ± 0.72	39.13 ± 4.19	0.15 ± 0.01	36.46 ± 2.96	34.93 ± 2.75	1.05 ± 0.08	3	1-2
*Aster altaicus* Willd.	14	10	4.02 ± 0.63	3.14 ± 0.36	2.87 ± 0.26	1.10 ± 0.11	3.67 ± 0.36	27.38 ± 0.93	0.13 ± 0.02	25.72 ± 1.98	26.97 ± 2.12	0.96 ± 0.07	2.5	2
*Erigeron lonchophyllus* Hook.	13	11	3.53 ± 0.64	2.70 ± 0.33	2.78 ± 0.30	0.97 ± 0.09	4.18 ± 0.38	26.68 ± 1.67	0.16 ± 0.01	23.95 ± 2.76	22.69 ± 1.72	1.06 ± 0.09	2	2
*Erigeron strigosus* Muhl. ex Willd.	24	18	2.47 ± 0.65	1.94 ± 0.27	2.08 ± 0.28	0.95 ± 0.14	3.16 ± 0.50	22.50 ± 1.54	0.14 ± 0.02	21.80 ± 1.22	20.81 ± 1.08	1.05 ± 0.05	2.5	1-2
*Erigeron acris* L.	20	16	2.96 ± 0.45	2.25 ± 0.24	2.58 ± 0.18	0.87 ± 0.09	3.72 ± 0.50	24.91 ± 1.83	0.15 ± 0.01	23.92 ± 1.54	23.02 ± 1.41	1.04 ± 0.05	2	1
*Erigeron acris* subsp. *politus* (Fr.) H. Lindb.	15	12	2.92 ± 0.25	2.27 ± 0.18	2.58 ± 0.25	0.89 ± 0.10	3.56 ± 0.35	24.30 ± 1.38	0.15 ± 0.01	23.59 ± 1.96	23.17 ± 1.37	1.02 ± 0.07	2	0-1

“Perforations at base” refers to the number of perforation rows (e.g., 1-2 rows) at the base of the spine.

Furthermore, for SEM analysis, standard polar and equatorial views of each species were selected. A 10 µm × 10 µm square grid was used to count the spines within, and the resulting trait parameters, termed Np and Ne, were used to characterize the distribution and number of spines in the polar and equatorial views, respectively. The counting rule was: ‘‘count the top, but not the bottom; count the left, but not the right.’ For these views, the mean values (M) and standard deviations (SD) of 10 pollen traits (P, E, P/E, T, L, T/L, D, H, D/H, Ss) were measured and calculated across the 21 representative species. Unlike the other traits, Np and Ne are presented as individual counts rather than M ± SD (**Table 2**; **Supplementary Data**).

### 2.3 Construction of the hierarchical dendrogram of pollen morphology

Pollen trait data were standardized using Z-scores (Andrade, 2021) to eliminate dimensional differences and ensure comparability. The data were then imported into IBM SPSS Statistics 26 (IBM Corp., Armonk, NY) for clustering analysis using Ward’s method and squared Euclidean distance. The proximity matrix was converted to Newick format using the “ape” and “readxl” packages in R v4.3.3 (https://www.R-project.org/). Visualization and refinement of the dendrogram were performed in Figtree v1.4.4, generating the hierarchical dendrogram of pollen morphology.

### 2.4 Construction of the ITS molecular phylogenetic tree

This study constructed a molecular phylogenetic tree based on ITS sequences from 21 species. Initially, the ITS sequences of these species were downloaded from the NCBI database, followed by quality control measures to ensure completeness and accuracy. Due to the temporary unavailability of ITS data for *Solidago altissima* in the NCBI database, *S. decurrens*, a congeneric species, was selected as a substitute for subsequent analyses. The ITS sequences were then aligned using MAFFT v.7.520 (Katoh and Standley, 2013) with default parameters to optimize sequence alignment. The aligned sequences were uploaded to the CIPRES Science Gateway platform (https://www.phylo.org/), where a maximum likelihood (ML) method was employed to construct the phylogenetic tree in RAxML (Stamatakis et al., 2008) under the GTR + GAMMA model, with 1,000 bootstrap replicates to enhance reliability. The resulting tree was visualized and adjusted in Figtree v1.4.4 (http://tree.bio.ed.ac.uk/software/figtree/), with branch modifications and annotations guided by the findings of Li et al. (2012) to produce the final phylogenetic tree. Throughout the adjustment process, clarity of the branches and integrity of the information were maintained to facilitate interpretation and presentation.

### 2.5 Data analysis and validation

Box plots of the 10 morphological traits measured under LM and SEM were generated using Excel 2019 (Microsoft Corp., Redmond, WA, USA). To further analyze differences in these morphological traits, an analysis of variance (ANOVA) was conducted on pollen morphological traits for all species using SPSS. Additionally, an independent samples t-test was conducted on pollen morphological data from *Aster* and *Erigeron* to compare morphological differences between these two genera.

In this study, the Robinson-Foulds (RF) distance method (Briand et al., 2020) was employed to compare the topological structures of the two phylogenetic trees. Initially, both trees were manually imported using a file browser for analysis. The RF distance between the trees was then calculated to assess their topological differences quantitatively. To enhance the interpretability of the results, we normalized the RF distance to produce the Tree Congruence Index (TCI), which quantifies the topological similarity between the two trees. A TCI value closer to 1 indicates higher topological similarity between the trees (de Vienne et al., 2007; Mir et al., 2013).

## 3 Results

### 3.1 Pollen morphological characteristics of the Astereae

**Figure 2:**
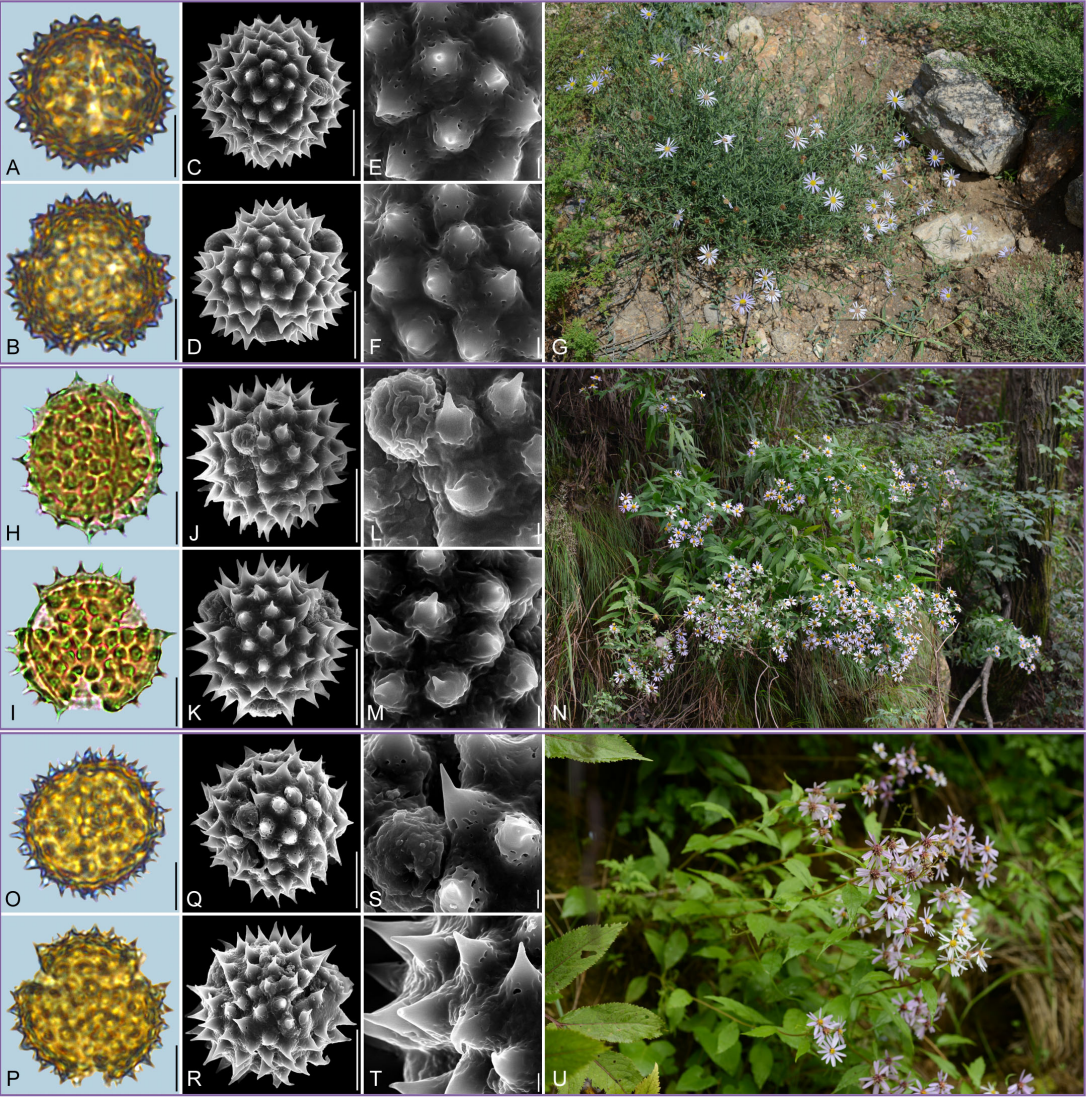
Pollen grains and the habitats of their source plants. **(A-G)** *Aster altaicus*; **(H-N)** *Aster ageratoides*; and **(O-U)** *Aster homochlamydeus*. Pollen grains in equatorial view under LM **(A, H, O)** and SEM **(C, E, J, L, Q, S)**, in polar view under LM **(B, I, P)** and SEM **(D, F, K, M, R, T)**, along with the habitats of their source plants (G cited from https://ppbc.iplant.cn/tu/10803110, last access: 6 November 2024, by ^©^ Y. S. Chen, N cited from https://ppbc.iplant.cn/tu/5937894, last access: 6 November 2024, by ^©^ R. **(B)** Zhu, U cited from https://ppbc.iplant.cn/tu/10697277, last access: 6 November 2024, by ^©^ Y. S. Chen). Scale bar in LM and SEM overview 10µm, and in SEM close-up 1µm.

**Figure 3:**
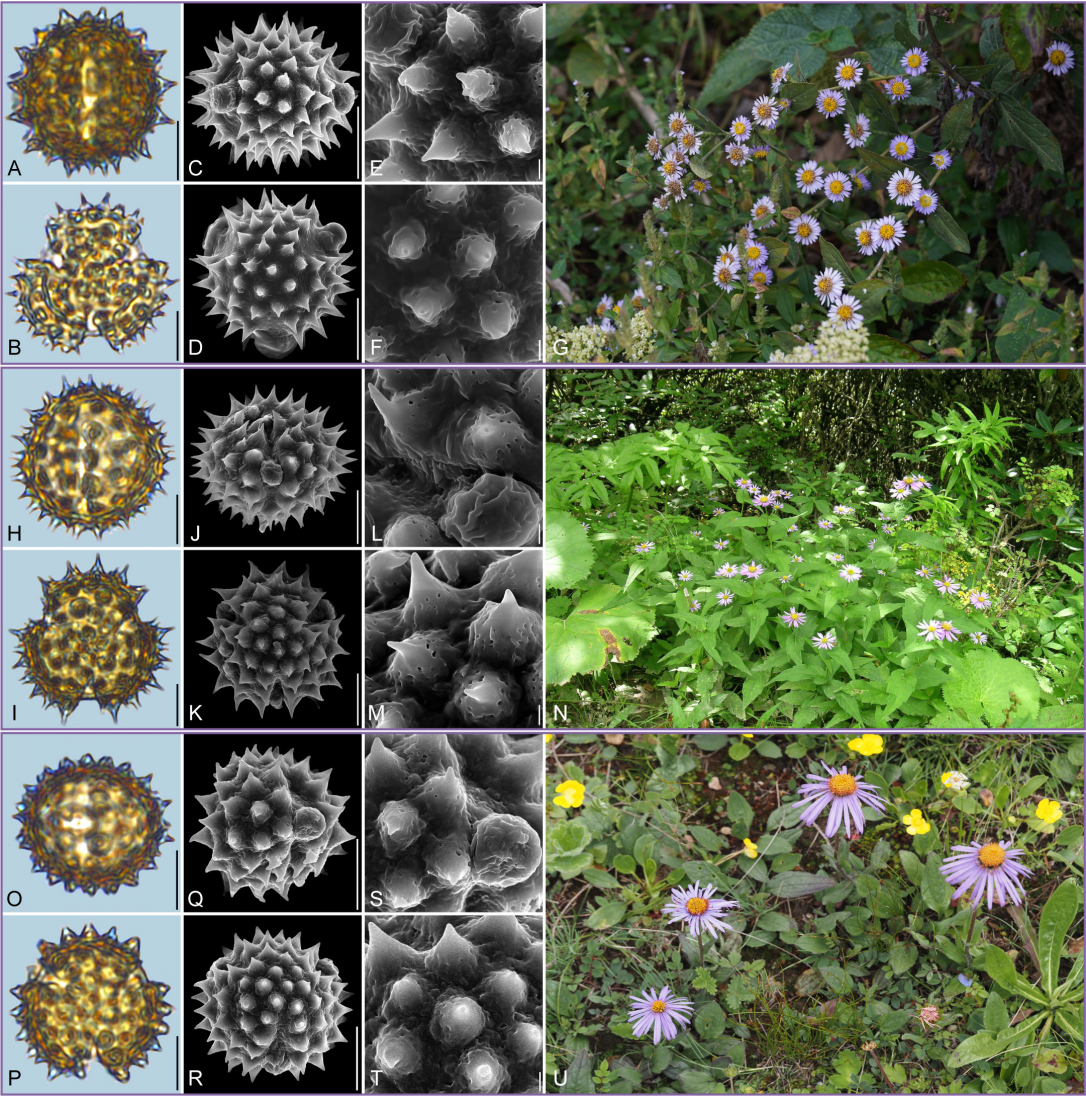
Pollen grains and the habitats of their source plants. **(A-G)** *Aster turbinatus*; **(H-N)** *Aster taliangshanensis*; and **(O-U)** *Aster brachytrichus*. Pollen grains in equatorial view under LM **(A, H, O)** and SEM **(C, E, J, L, Q, S)**, in polar view under LM **(B, I, P)** and SEM **(D, F, K, M, R, T)**, along with the habitats of their source plants (G cited from https://ppbc.iplant.cn/tu/15652366, last access: 6 November 2024, by ^©^ X. Y. Ye, N cited from https://ppbc.iplant.cn/tu/451749, last access: 6 November 2024, by ^©^ Y. S. Chen, U cited from https://ppbc.iplant.cn/tu/15002989, last access: 6 November 2024, by ^©^ Y. S. Chen). Scale bar in LM and SEM overview 10µm, and in SEM close-up 1µm.

**Figure 4:**
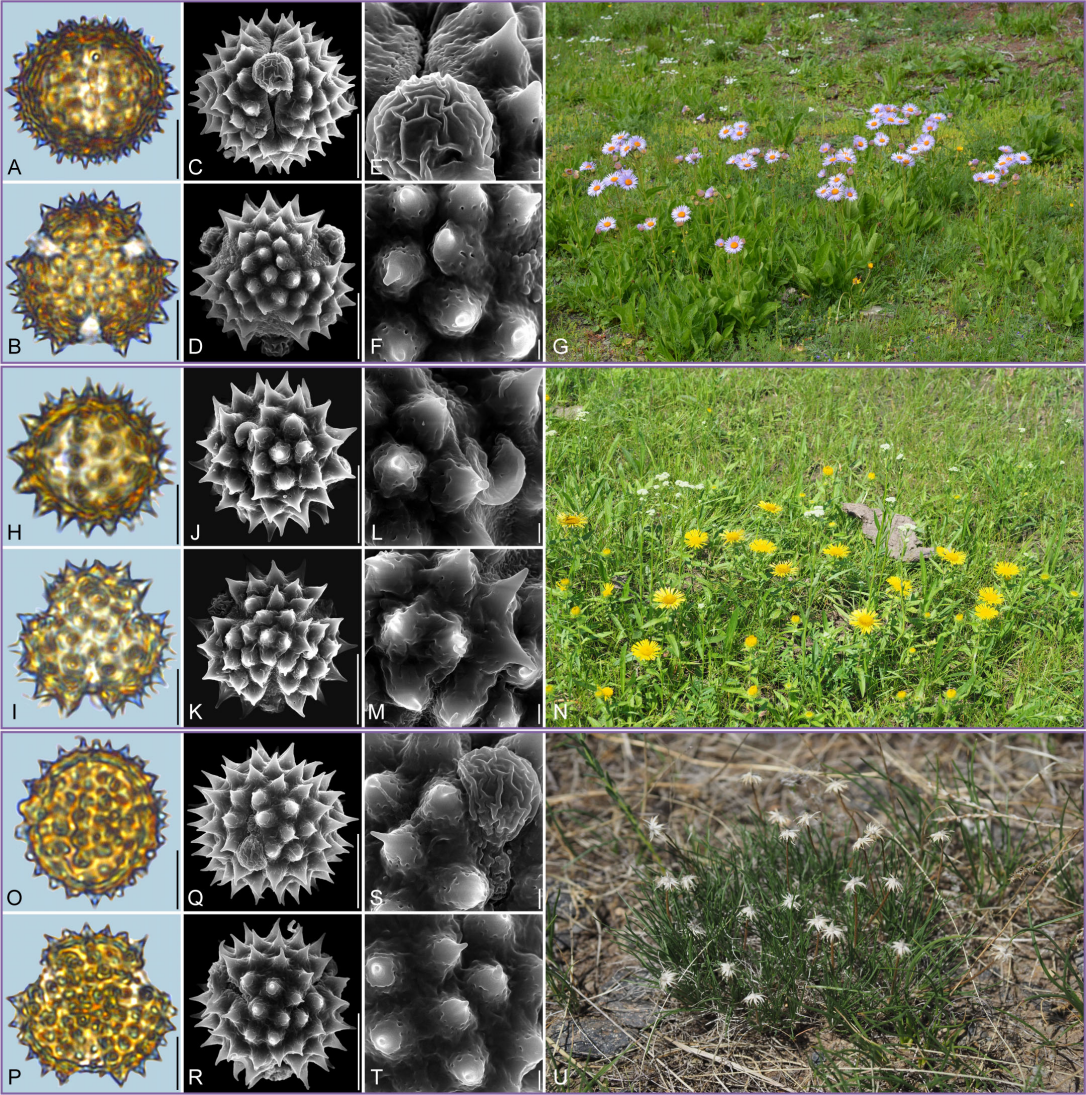
Pollen grains and the habitats of their source plants. **(A-G)** *Aster yunnanensis*; **(H-N)** *Turczaninovia fastigiata*; and **(O-U)** *Arctogeron gramineum*. Pollen grains in equatorial view under LM **(A, H, O)** and SEM **(C, E, J, L, Q, S)**, in polar view under LM **(B, I, P)** and SEM **(D, F, K, M, R, T)**, along with the habitats of their source plants (G cited from https://ppbc.iplant.cn/tu/11716277, last access: 6 November 2024, by ^©^ Y. P. Zeng, N cited from https://ppbc.iplant.cn/tu/8233925, last access: 6 November 2024, by ^©^ Q. W. Lin, U cited from https://ppbc.iplant.cn/tu/8258978, last access: 6 November 2024, by ^©^ Q. W. Lin). Scale bar in LM and SEM overview 10µm, and in SEM close-up 1µm.

**Figure 5:**
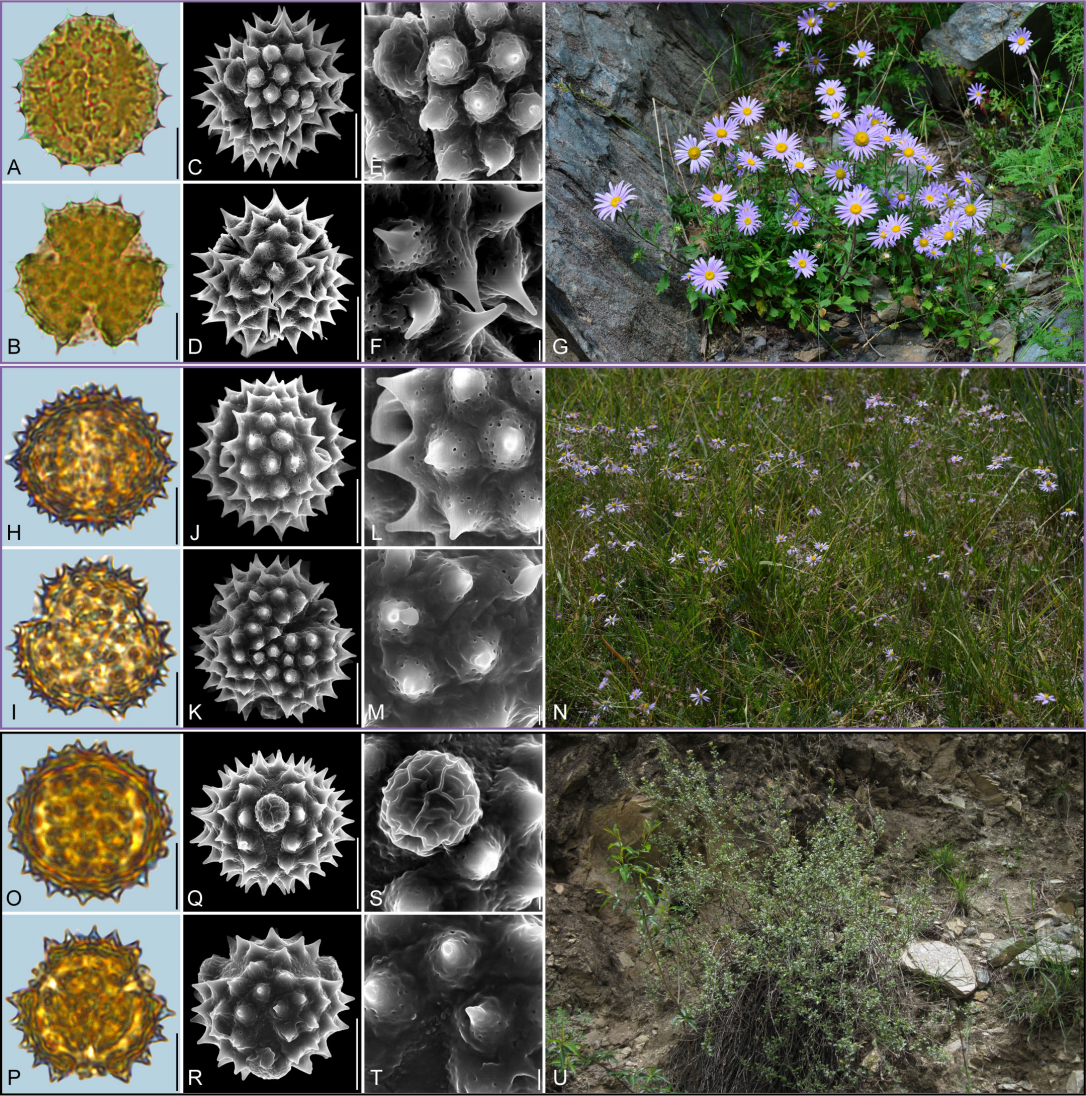
Pollen grains and the habitats of their source plants. **(A-G)** *Callistephus chinensis*; **(H-N)** *Galatella angustissima*; and **(O-U)** *Formania mekongensis*. Pollen grains in equatorial view under LM **(A, H, O)** and SEM **(C, E, J, L, Q, S)**, in polar view under LM **(B, I, P)** and SEM **(D, F, K, M, R, T)**, along with the habitats of their source plants (G cited from https://ppbc.iplant.cn/tu/2409876, last access: 6 November 2024, by ^©^ R. **(B)** Zhu, N cited from https://ppbc.iplant.cn/tu/10824525, last access: 6 November 2024, by ^©^ Y. S. Chen, U cited from https://ppbc.iplant.cn/tu/836417, last access: 6 November 2024, by ^©^ Y. S. Chen). Scale bar in LM and SEM overview 10µm, and in SEM close-up 1µm.

**Figure 6:**
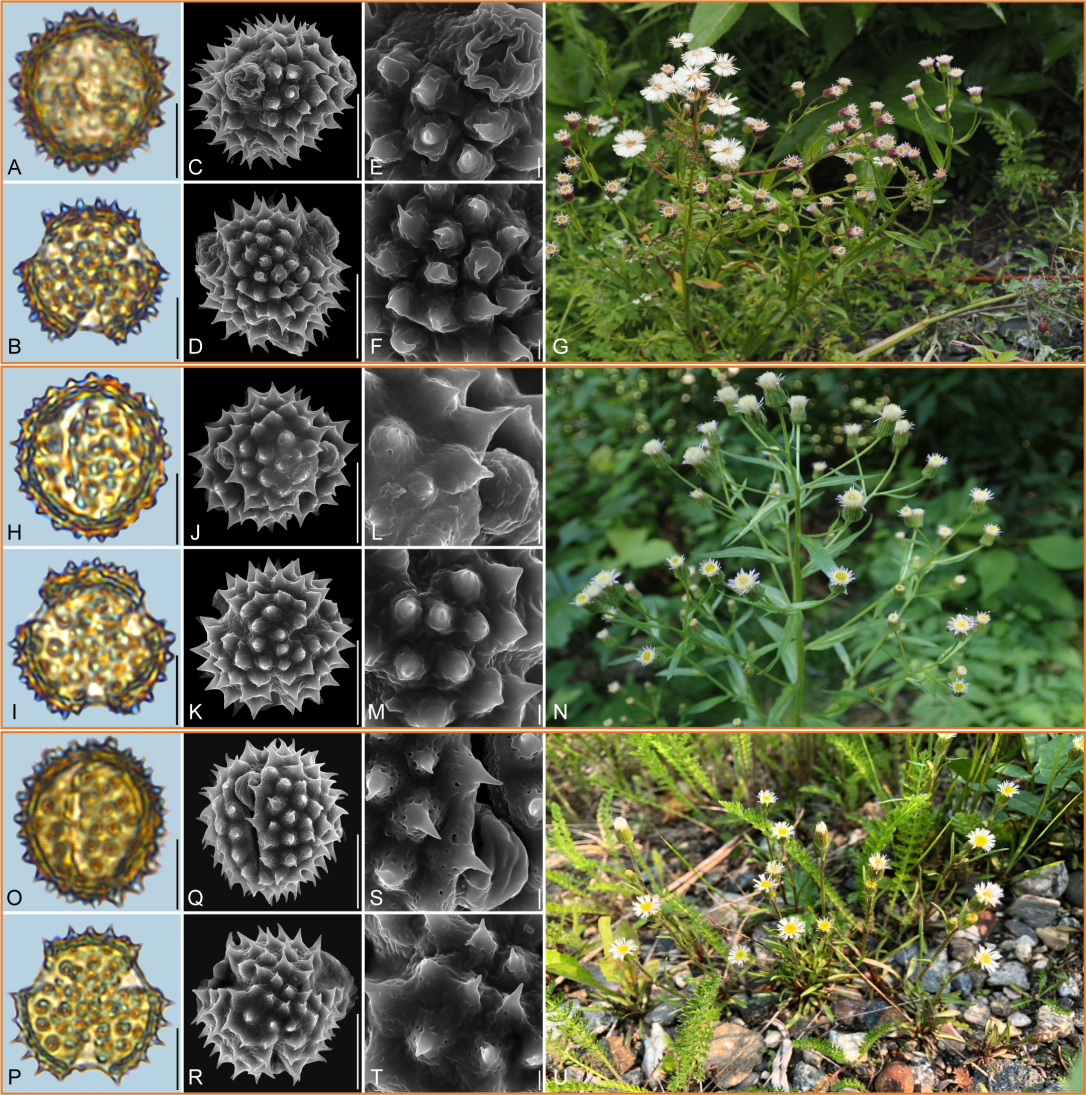
Pollen grains and the habitats of their source plants. **(A-G)** *Erigeron acris*; **(H-N)** *Erigeron acris* subsp. *politus*; and **(O-U)** *Erigeron lonchophyllus*. Pollen grains in equatorial view under LM **(A, H, O)** and SEM **(C, E, J, L, Q, S)**, in polar view under LM **(B, I, P)** and SEM **(D, F, K, M, R, T)**, along with the habitats of their source plants (G cited from https://ppbc.iplant.cn/tu/15006256, last access: 6 November 2024, by ^©^ Y. S. Chen, N cited from https://ppbc.iplant.cn/tu/8196316, last access: 6 November 2024, by ^©^ Q. W. Lin, U cited from https://www.inaturalist.org/observations/28542542, last access: 6 November 2024, by ^©^ J. Grant). Scale bar in LM and SEM overview 10µm, and in SEM close-up 1µm.

**Figure 7:**
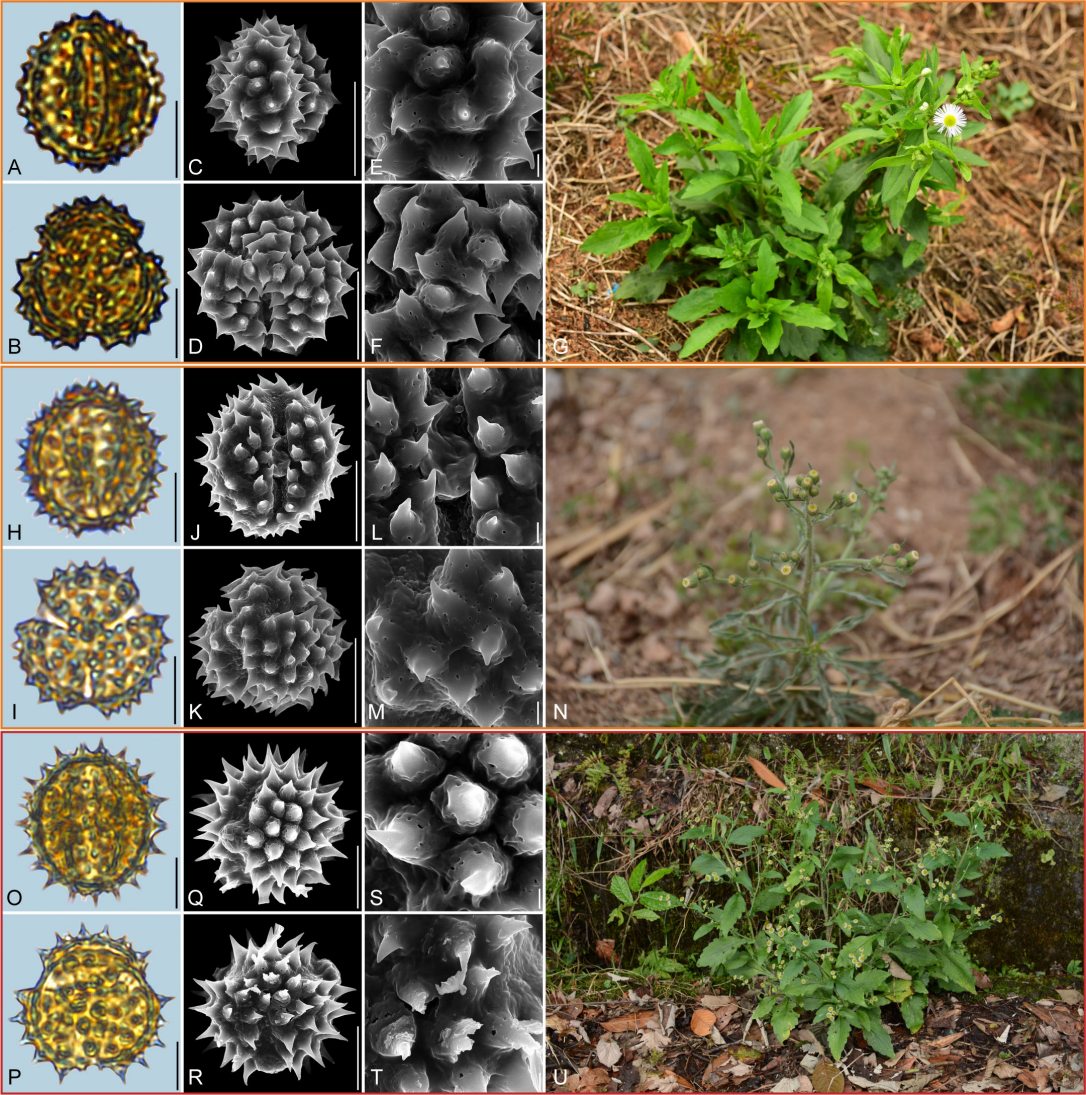
Pollen grains and the habitats of their source plants. **(A-G)** *Erigeron strigosus*; **(H-N)** *Eschenbachia japonica*; and **(O-U)** *Myriactis wallichii*. Pollen grains in equatorial view under LM **(A, H, O)** and SEM **(C, E, J, L, Q, S)**, in polar view under LM **(B, I, P)** and SEM **(D, F, K, M, R, T)**, along with the habitats of their source plants (G cited from https://ppbc.iplant.cn/tu/7206727, last access: 6 November 2024, by ^©^ A. Liu, N cited from https://ppbc.iplant.cn/tu/11445230, last access: 6 November 2024, by ^©^ Y. P. Zeng, U cited from https://ppbc.iplant.cn/tu/11461423, last access: 6 November 2024, by ^©^ Y. P. Zeng). Scale bar in LM and SEM overview 10µm, and in SEM close-up 1µm.

**Figure 8:**
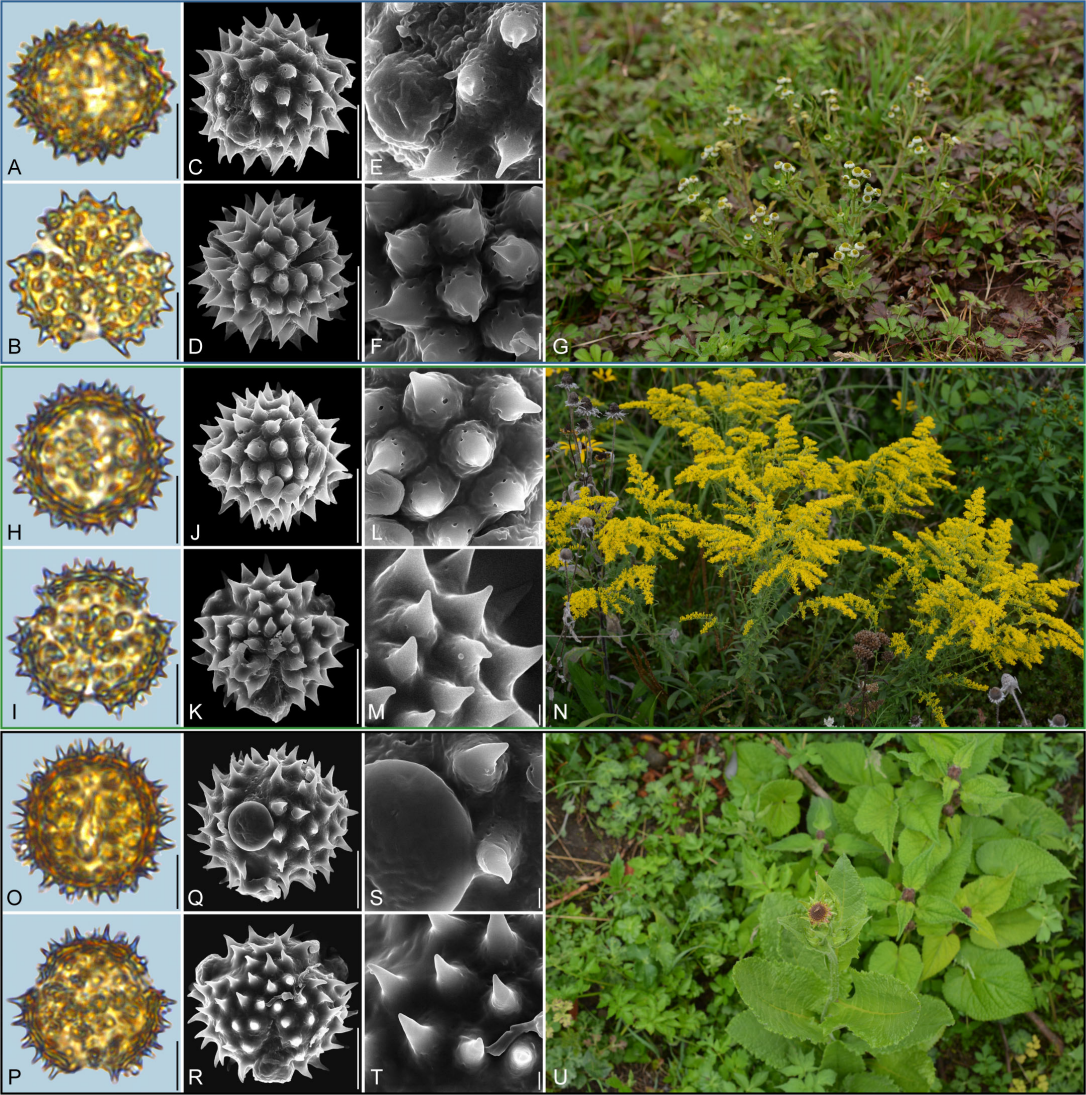
Pollen grains and the habitats of their source plants. **(A-G)** *Dichrocephala benthamii*; **(H-N)** *Solidago altissima*; and **(O-U)** *Nannoglottis carpesioides*. Pollen grains in equatorial view under LM **(A, H, O)** and SEM **(C, E, J, L, Q, S)**, in polar view under LM **(B, I, P)** and SEM **(D, F, K, M, R, T)**, along with the habitats of their source plants (G cited from https://ppbc.iplant.cn/tu/11457706, last access: 6 November 2024, by ^©^ Y. P. Zeng, N cited from https://ppbc.iplant.cn/tu/10518902, last access: 6 November 2024, by ^©^ Y. S. Chen, U cited from https://ppbc.iplant.cn/tu/11485800, last access: 6 November 2024, by ^©^ Y. P. Zeng). Scale bar in LM and SEM overview 10µm, and in SEM close-up 1µm.

**Figure 9:**
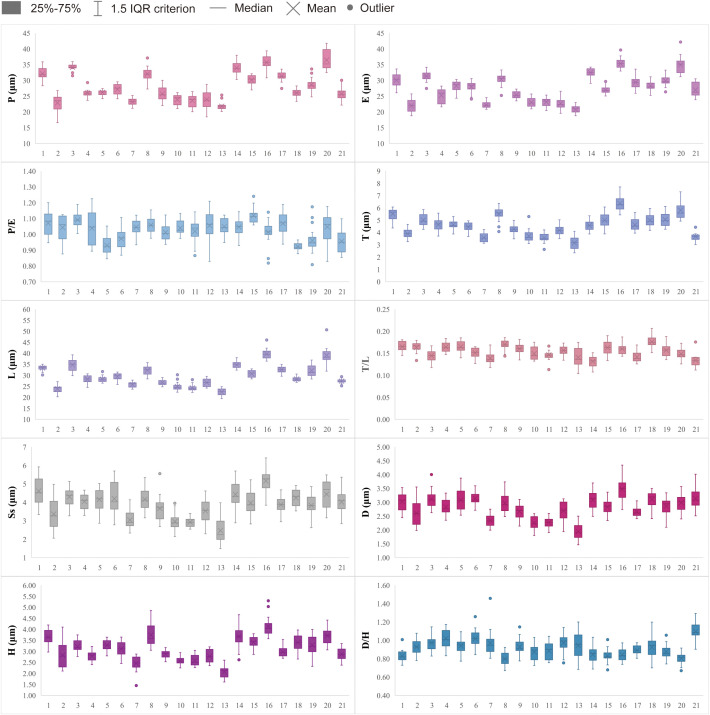
Boxplots of 21 sampled taxa showing the variations in pollen morphological traits. 1. *Nannoglottis carpesioides*; 2. *Dichrocephala benthami*i; 3. *Galatella angustissima*; 4. *Formania mekongensis*; 5. *Aster yunnanensis*; 6. *Aster brachytrichus*; 7. *Eschenbachia japonica*; 8. *Myriactis wallichii*; 9. *Solidago altissima*; 10. *Erigeron acris*; 11. *Erigeron acris* subsp. *Politus*; 12. *Erigeron lonchophyllus*; 13. *Erigeron strigosus*; 14. *Callistephus chinensis*; 15. *Arctogeron gramineum*; 16. *Aster taliangshanensis*; 17. *Aster ageratoides*; 18. *Turczaninovia fastigiata*; 19. *Aster turbinatus*; 20. *Aster homochlamydeus*; 21. *Aster altaicus*.

Detailed pollen morphological data observed under LM and SEM, along with habitat information for the 21 sampled species, are presented in **Figures 2**–**8**. **Table 2** summarizes the quantitative values of pollen morphological traits for these species. Except for Np and Ne traits, the remaining 10 morphological characteristics are expressed as mean ± standard deviation (M ± SD). **Table 3** presents the qualitative morphological traits of pollen, providing an overview of its key characteristics. Box plots (**Figure 9**) depict the distribution patterns of these data, highlighting the interquartile range (25%-75%). The specific trait information for each species is detailed in the **Supplementary Material**.

**Table 3:** Qualitative morphological traits of pollen in 21 selected species.

Species	Pollen shape	Colporus	Exine sculpturing (LM)	Exine sculpturing (SEM)	Aperture membrane	Spine shape	Inter-spinal
*Arctogeron gramineum* (L.) DC.	subprolate	tricolporate	spinose	echinate	granulate	tapering to a sharp, pointed tip	perforate
*Callistephus chinensis* (L.) Nees	spheroidal	tricolporate	spinose	echinate	granulate	base expanded, tapering to a sharp tip	perforate
*Eschenbachia japonica* (Thunb.) J. Kost.	spheroidal	tricolporate	spinose	echinate	granulate	tapering to a sharp	perforate
*Dichrocephala benthamii* C. B. Clarke	spheroidal	tricolporate	spinose	echinate	granulate	tapering to a sharp	perforate
*Formania mekongensis* W.W.Sm. & J.Small	spheroidal	tricolporate	spinose	echinate	granulate	tapering to a sharp	perforate
*Galatella angustissima* (Tausch) Novopokr.	spheroidal	tricolporate	spinose	echinate	granulate	tapering to a sharp	perforate
*Myriactis wallichii* Less.	spheroidal	tricolporate	spinose	echinate	granulate	tapering to a sharp, pointed tip	perforate
*Nannoglottis carpesioides* Maxim.	spheroidal	tricolporate	spinose	echinate	granulate	tapering to a sharp	perforate
*Solidago altissima* L.	spheroidal	tricolporate	spinose	echinate	granulate	tapering to a sharp	perforate
*Turczaninovia fastigiata* (Fisch.) DC.	spheroidal	tricolporate	spinose	echinate	granulate	tapering to a sharp, pointed tip	perforate
*Aster ageratoides* Turcz.	spheroidal	tricolporate	spinose	echinate	granulate	tapering to a sharp, pointed tip	perforate
*Aster yunnanensis* Franch.	spheroidal	tricolporate	spinose	echinate	granulate	tapering to a sharp	perforate
*Aster brachytrichus* Franch.	spheroidal	tricolporate	spinose	echinate	granulate	tapering to a sharp	perforate
*Aster taliangshanensis* Y. Ling	spheroidal	tricolporate	spinose	echinate	granulate	tapering to a sharp, pointed tip	perforate
*Aster turbinatus* S. Moore	spheroidal	tricolporate	spinose	echinate	granulate	tapering to a sharp	perforate
*Aster homochlamydeus* Hand.-Mazz.	spheroidal	tricolporate	spinose	echinate	granulate	tapering to a sharp	perforate
*Aster altaicus* Willd.	spheroidal	tricolporate	spinose	echinate	granulate	base expanded, tapering to a point	perforate
*Erigeron lonchophyllus* Hook.	spheroidal	tricolporate	spinose	echinate	granulate	tapering to a sharp, pointed tip	perforate
*Erigeron strigosus* Muhl. ex Willd.	spheroidal	tricolporate	spinose	echinate	granulate	tapering to a sharp, pointed tip	perforate
*Erigeron acris* L.	spheroidal	tricolporate	spinose	echinate	granulate	tapering to a sharp, pointed tip	perforate
*Erigeron acris* subsp. *politus* (Fr.) H. Lindb.	spheroidal	tricolporate	spinose	echinate	granulate	tapering to a sharp	perforate

#### 3.1.1 Pollen shape and apertures

The results indicate that most pollen grains of the studied species are nearly spheroidal (0.90 < P/E < 1.10), with only *Arctogeron gramineum* exhibiting a subprolate shape (P/E = 1.12 > 1.10) (**Figure 4**). All species display three-colporate apertures, which are clearly observed as tricolporate structures under both LM and SEM. The P/E (the length of polar axis/the length of equatorial axis) ranges from 0.92 to 1.12. In equatorial view, P ranges from 21.80 to 36.46 µm, while E ranges from 20.81 to 35.32 µm. Significant interspecies differences were observed in P, E, and P/E (p < 0.01).

#### 3.1.2 Pollen exine ornamentation

All pollen grains exhibit spines, which are prominently spinose under SEM. The spines gradually taper, typically conical in shape, or have a noticeably widened base. The D ranges from 1.94 to 3.48 µm, the H ranges from 2.08 to 4.14 µm, and the D/H (diameter of spinule base/spinule height) ranges from 0.80 to 1.10. Significant interspecies differences were observed in D and H (p < 0.01). Tiny pores are present at the spinule bases, with 1-3 layers that vary depending on species and individual differences.

### 3.2 Pollen traits and clustering results of the Astereae

**Figure 10:**
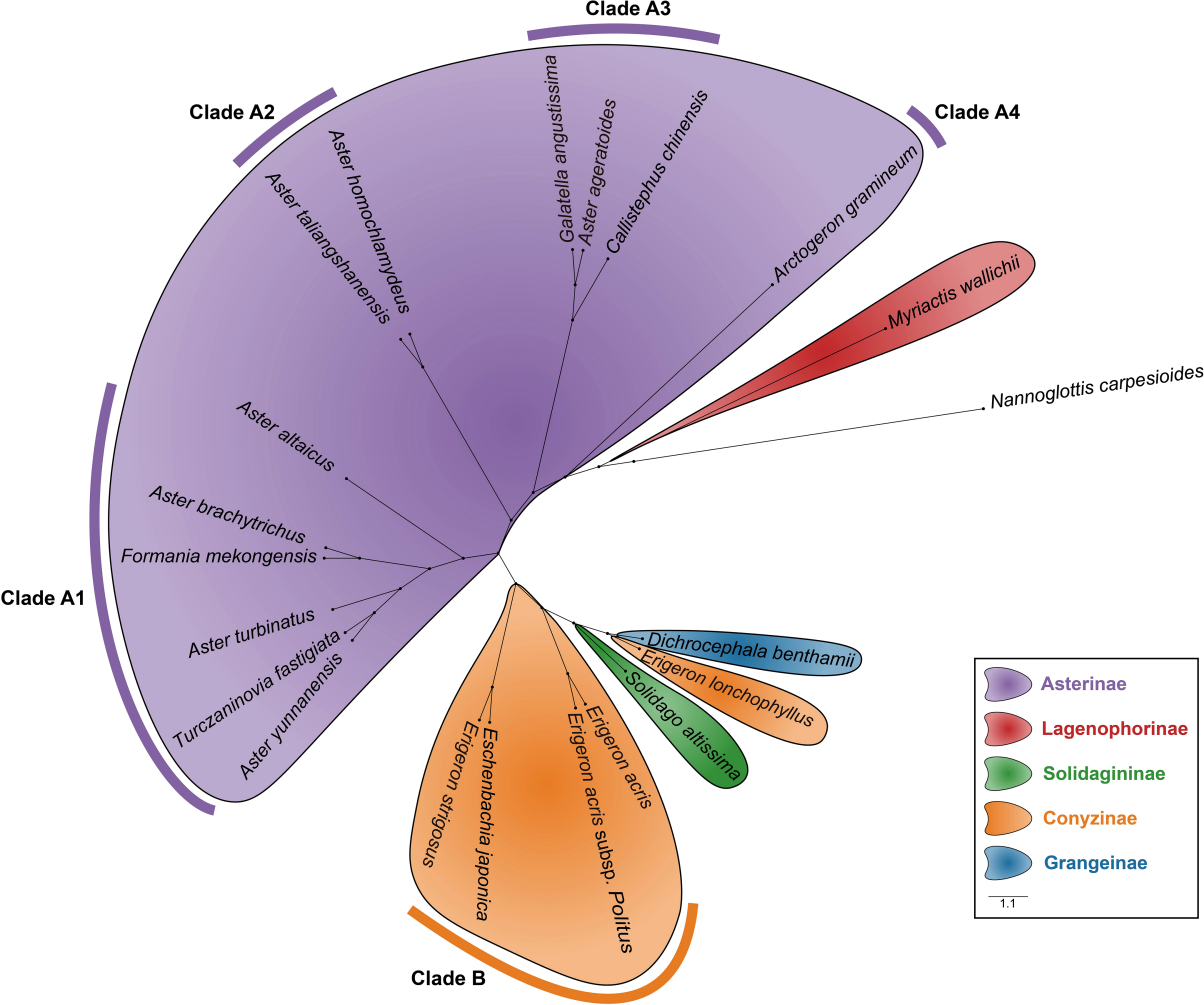
The hierarchical dendrogram of pollen morphology depicts the classification of pollen types within the Astereae based on morphological features.

Morphological traits, including pollen size, aperture type, and exine ornamentation, were measured for 21 species of Astereae. Based on these data, a hierarchical dendrogram of pollen morphology was constructed (**Figure 10**). *Nannoglottis carpesioides* was used as the outgroup, following the phylogenetic framework proposed by Li et al. (2012). The clustering results revealed that species within the same subtribe formed distinct, well-defined clusters, with clear separation between subtribes. At the genus level, *Aster* and *Erigeron* were grouped into well-separated branches, reflecting differentiation between the two genera.

Based on the clustering results, the branches corresponding to the Asterinae and Conyzinae were designated as Clade A and Clade B, respectively. Clade A was further subdivided into four branches: Clade A1, Clade A2, Clade A3, and Clade A4 (**Figure 10**). Principal component analysis (PCA) of the 21 Astereae species identified eight key pollen morphological traits—Ss, E, D, L, H, Np, P, and T — that distinguished these clusters. The results of the t-test for these traits are presented in **Table 4**.

**Table 4:** The t-test analysis results for the pollen morphological characteristics of the *Aster* L. and the *Erigeron* L.

Pollen morphological characters	Aster L.	Erigeron L.
Ss (μm)	significant	significant
D (μm)	significant	significant
H (μm)	significant	significant
D/H	non-significant	non-significant
T (μm)	significant	significant
L (μm)	significant	significant
T/L	non-significant	non-significant
P (μm)	significant	significant
E (μm)	significant	significant
P/E	significant	significant

Several pollen traits partially explain the differences between the pollen types of *Aster* and *Erigeron*. The L values of *Aster* pollen (Clade A1 and Clade A2) range from 27.38 to 39.72 μm, significantly larger than those of *Erigeron* (Clade B), which range from 22.50 to 26.76 μm (t-test, p < 0.01). This trait serves as a reliable distinguishing feature for the latter. Within *Aster*, Clade A2 (*A. taliangshanensis* and *A. homochlamydeus*) exhibits higher L values (39.72 μm and 39.13 μm, respectively) compared to Clade A1 (27.38-32.17 μm). Similarly, The Ss shows significant differences between the two genera (*Aster*: 3.83-5.16 μm; *Erigeron*: 2.47-3.67 μm; t-test, p < 0.01), making it another critical parameter for differentiation. In contrast, the Np and Ne values are generally higher in *Erigeron*, reflecting a denser spine distribution compared to *Aster*. Regarding D and H, *Erigeron* exhibits smaller values (D: 1.94-2.70 μm; H: 2.08-2.88 μm) than *Aster* (D: 2.83-3.48 μm; H: 2.78-4.14 μm). These differences highlight the short and narrow spines in *Erigeron*, in contrast to the long and wide spines in *Aster*. Additionally, pollen grains of *Erigeron* observed in polar view under LM are significantly smaller than those of *Aster*.

Meanwhile, some species exhibit unique characteristics. For instance, *A. ageratoides*, grouped within Clade A3, shares most of its pollen traits with other *Aster* species. However, its D value (2.65 μm) is lower than the minimum observed in other *Aster* species, while its P/E (1.07) exceeds their maximum. Similarly, *Arctogeron gramineum* displays the highest P/E (1.12), indicating a pollen shape approaching subprolate. Additionally, *Myriactis wallichii* (Lagenophorinae), *Solidago decurrens* (Solidagininae), and *Dichrocephala benthamii* (Grangeinae), each cluster within distinct branches corresponding to their respective subtribes, reflecting clear subtribal-level separation.

### 3.3 ITS molecular phylogenetic tree of the Astereae

**Figure 11:**
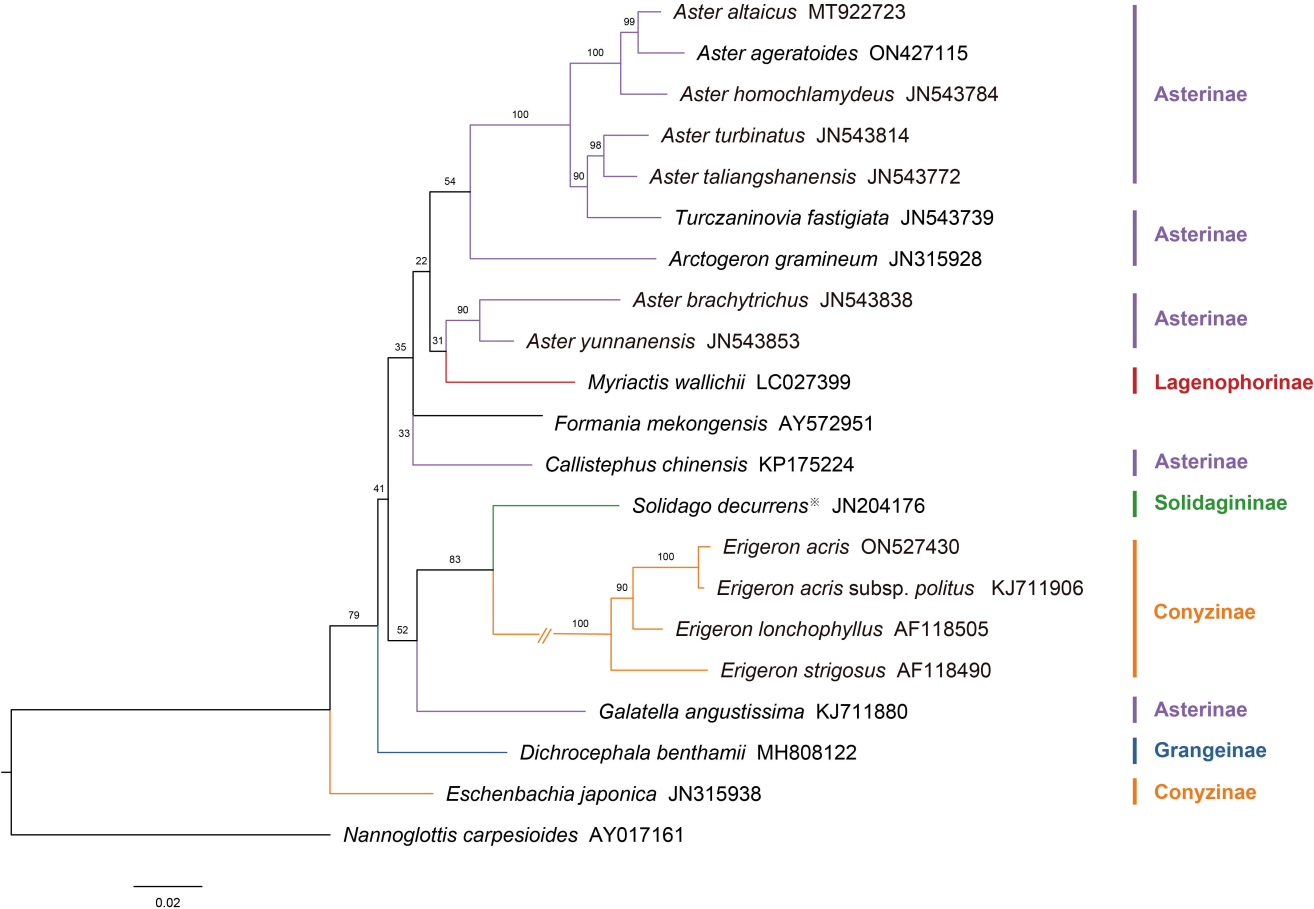
The molecular phylogeny tree of the Astereae is based on nuclear ribosomal DNA internal transcribed spacer sequences. Subtribus classification follows the framework of Anderberg et al. (2007) as outlined. (Note: ※*Solidago decurrens* replaces *S. altissima*).

A molecular phylogenetic tree was constructed based on ITS sequence data from 21 Astereae species (**Figure 11**), following the framework proposed by Li et al. (2012). *N. carpesioides*, identified as basal or near-basal within the Astereae (Liu et al., 2002), was selected as the outgroup. This tree illustrates the phylogenetic relationships among the studied species and serves as a basis for comparison with the pollen morphology dendrogram.

The molecular phylogenetic tree reveals the evolutionary relationships within the Astereae. Asterinae species form a major branch, with closely clustered representatives such as *A. taliangshanensis*, *A. homochlamydeus*, and *A. altaicus*. Conyzinae is represented by *Erigeron* species (e.g., *E. acris*, *E. lonchophyllus*), forming a distinct lineage, while *Eschenbachia japonica* appears as a separate branch within the subtribe. Other subtribes, such as Lagenophorinae (*M. wallichii*), Solidagininae (*S. decurrens*), and Grangeinae (*D. benthamii*), each form independent branches, reflecting their phylogenetic distinctiveness.

### 3.4 Comparison of molecular phylogeny and pollen morphology dendrogram in the Astereae

The TCI value between the pollen morphology dendrogram and the molecular phylogenetic tree was 0.545.

At the subtribal level, the pollen morphology dendrogram revealed well-defined clustering patterns. Species of Asterinae were primarily grouped in Clade A (**Figure 10**), whereas their distribution in the molecular phylogenetic tree was more dispersed. Notably, although *Erigeron* species within Conyzinae clustered together in a single branch in the molecular tree, *E. lonchophyllus* was separated from the main cluster of *Erigeron* species in the pollen dendrogram. Other subtribes, including Lagenophorinae, Solidagininae, and Grangeinae, formed independent branches in both trees.

At the genus level, species of *Aster* and *Erigeron* showed consistent clustering patterns in both trees. Species of *Aster* (e.g., *A. altaicus*, *A. turbinatus*, *A. homochlamydeus*, and *A. taliangshanensis*) clustered within Clades A1 and A2 in the pollen dendrogram, closely matching their distribution in the molecular phylogenetic tree. Similarly, species of *Erigeron* (e.g., *E. acris* and *E. strigosus*) formed distinct major branches in both trees, reinforcing their phylogenetic independence. In contrast, genera such as *Callistephus* and *Solidago* showed lower congruence between the two trees. Notably, *F. mekongensis* clustered with Asterinae species in Clade A1 (**Figure 10**), but appeared on a neighboring branch in **Figure 11**.

## 4 Discussion

### 4.1 The significance of pollen morphology in the systematic classification of the Astereae

The TCI value of 0.545 indicates a moderate topological similarity (de Vienne et al., 2007; Mir et al., 2013) between the molecular and morphological trees, which is expected given the different data types used. While some differences are inevitable, this cross-validation strongly supports the use of pollen morphology in classification (Keating et al., 2023). The pollen morphology clustering tree clearly shows species groupings within the same subtribe, highlighting its effectiveness. Lagenophorinae and Grangeinae form independent branches, distinct from Asterinae and Conyzinae. These findings underscore the significance of pollen traits in subtribal classifications and phylogenetic studies (Moon et al., 2008). Moreover, the high concordance with the macroscopic morphological classification framework (Anderberg et al., 2007) and the molecular phylogenetic tree (Li et al., 2012) reinforces the reliability of pollen morphology in subtribal-level classification. At the genus level, variations in pollen traits reflect phylogenetic relationships and distinctions among genera, highlighting their unique evolutionary trends and affinities with closely related taxa (Wodehouse, 1935; Zhang and Zhou, 2016).

*N. carpesioides* occupies the basal position of the pollen morphology dendrogram (**Figure 10**), showing a trend of decreasing pollen size as species radiate outward. Within Asterinae, species in Clades A2, A3, and A4 exhibit larger pollen parameters (P and E) compared to Clade A1. In contrast, Conyzinae species consistently display smaller P and E values. These findings provide important insights into the phylogenetic relationships within Astereae, particularly the separation of *Erigeron* from Asterinae, which aligns more closely with Conyzinae (Zhang and Zhou, 2016; Iamonico, 2018; Bhattacharjee et al., 2024; Chakraborty et al., 2024). Interestingly, *Turczaninovia fastigiata* (P/E = 0.92) and *Arctogeron gramineum* (P/E = 1.12) cluster closely in **Figure 11**, yet are positioned at opposite ends of Asterinae (Clade A) in **Figure 10**. This discrepancy may be attributed to their P/E values representing the minimum and maximum observed in this study, suggesting that the P/E may be an important factor influencing pollen morphology clustering (Wodehouse, 1928; Bahadur et al., 2022).

The taxonomic placement of *F. mekongensis* remains unresolved. Shi and Fu (1983) classified it within Chrysantheminae of Anthemideae, while Chen and Brouillet (2011b) considered its classification uncertain. Fu et al. (2016) placed it in Astereae based on molecular phylogenetic analysis, and Nesom (2020) later assigned it to the newly established subtribe Formaniinae within Astereae. In this study, pollen morphological clustering places *F. mekongensis* with Asterinae species in Clade A1 (**Figure 10**), providing the first palynological evidence supporting its placement in Asterinae. This finding builds on earlier studies that recognized *F. mekongensis* within Astereae (Fu et al., 2016).

In summary, pollen morphological analysis reveals significant phylogenetic patterns and evolutionary trends across taxonomic levels. Unlike molecular methods, it offers unique structural insights and visual evidence (Wodehouse, 1928, 1929; Keating et al., 2023). This study underscores the value of pollen morphology in subtribal classification within Astereae, helps distinguish between the *Aster* and *Erigeron*, and provides new insights into the taxonomic placement of *F. mekongensis*. Although focused on Astereae, the approach presented here has broader implications for using pollen traits in plant systematics. By integrating molecular and morphological data, this work paves the way for more comprehensive plant classification at various taxonomic levels and encourages future research into combining these data types.

### 4.2 Taxonomic significance of pollen morphology in *Aster* and *Erigeron*

*Aster*, the largest genus in Astereae, is of considerable economic importance. Its capitula are typically solitary or arranged in corymbiform or paniculiform synflorescences (Chen et al., 2011, 2024). *Erigeron*, the second-largest genus in the tribe, is characterized by radiate capitula (Chen and Brouillet, 2011a; Zhang and Zhou, 2016). Despite these differences, morphological similarities between certain species of *Aster* and *Erigeron* have complicated their classification (Nesom, 1994; Li et al., 2012; Fu et al., 2016). This study identifies significant differences in pollen size and exine ornamentation between the two genera, offering new insights into their taxonomic distinction.

SEM reveals differences in exine ornamentation, with *Aster* pollen exhibiting long, broad, and sparsely distributed spines, while *Erigeron* pollen features short, narrow, and more densely arranged spines. Under LM, the pollen grains of *Aster* are significantly larger than those of *Erigeron*, with nearly a twofold difference in size. Despite these morphological differences, both genera share a typical spinulose ornamentation pattern (Skvarla et al., 1977; Zhang and Zhou, 2016). Zhang and Zhou (2016) reported a close relationship between *E. strigosus*, *A. batangensis*, and *T. fastigiata*. Chen et al. (2024) further demonstrated that *A. batangensis* and *A. yunnanensis* cluster within the “Alpine *Aster*” group, supporting their recognition as a distinct taxonomic unit. In our phylogenetic analysis (**Figure 10**), *A. yunnanensis* and *T. fastigiata* closely cluster within Clade A1, showing a phylogenetic affinity with *E. strigosus* in Clade B. These findings corroborate previous studies and, coupled with the observed pollen morphological differences, highlight the complex evolutionary relationships among these taxa.

From a biogeographical perspective, *Aster* and *Erigeron* belong to the Eurasian (EA) and North American (NA) evolutionary lineages of the Astereae, respectively (Li et al., 2012). Molecular phylogenetic studies have revealed a significant genetic divergence between the EA and NA lineages (Noyes and Rieseberg, 1999; Selliah and Brouillet, 2008; Brouillet et al., 2009; Li et al., 2012; Jafari et al., 2015; Korolyuk et al., 2015). The observed differences in pollen morphology likely reflect divergent natural selection pressures as the two lineages adapted to distinct ecological environments (Wang and Wang, 1983). *Aster* has diversified in temperate climates, exhibiting high species diversity (Chen et al., 2011, 2024). In contrast, *Erigeron* has adapted to arid environments, where the role of insect pollination is reduced, leading to changes in pollen size and other traits, such as spine reduction, as part of the adaptation to these conditions (Zhang and Zhou, 2016; Zhang et al., 2019; Bhattacharya et al., 2022). These ecological adaptations, reflected in pollen morphology, are also supported by molecular phylogenetic evidence (Li et al., 2012; Jafari et al., 2015).

### 4.3 Roles and prospects of pollen morphology in Asteraceae phylogenetics

Pollen morphology, combining the strengths of both morphological and molecular analyses, provides an accurate and cost-effective tool for plant taxonomy (Kriebel et al., 2017; Keating et al., 2023). In the Astereae, pollen morphology analyses of *Aster* and *Erigeron* reveal significant differences in pollen grain size and exine spine morphology. These microstructural traits offer reliable evidence for taxonomic classification and are essential for elucidating the phylogenetic relationships within the Astereae. The application of pollen morphology is highly operable and reliable, overcoming the limitations of single-method morphological or molecular studies, and serves as a critical complement to systematic plant taxonomy (Kriebel et al., 2017; Wang et al., 2023).

Future research on pollen morphology in Asteraceae may focus on the following aspects: (1) employing high-resolution imaging techniques, such as SEM and transmission electron microscopy (TEM), to conduct detailed analyses of pollen microstructures, uncovering subtle interspecific differences and improving classification accuracy (Polevova et al., 2023; Gabarayeva et al., 2024); (2) integrating molecular markers, such as ribosomal DNA and chloroplast DNA, to further explore genetic diversity and phylogenetic relationships within the Asteraceae (Zhang et al., 2024); and (3) expanding sample sizes and ecological ranges, thereby deepening our understanding of the role of pollen in ecological adaptation and evolutionary processes (Martín-Hernanz et al., 2019; Cozzolino et al., 2021).

## 5 Conclusions

This study integrates molecular systematics and pollen morphology to examine phylogenetic relationships within Astereae. The alignment of pollen morphology with molecular phylogenetic trees demonstrates that palynology is a reliable tool for plant taxonomy at both the genus and subtribal levels. Significant morphological differences were observed between *Aster* and *Erigeron*, and the placement of *F. mekongensis* provides further evidence for its taxonomic position. These findings highlight the potential of pollen data to refine classification and clarify evolutionary relationships within Astereae. The integration of palynological and molecular data offers a comprehensive approach to plant systematics. Future studies incorporating broader taxon sampling, additional molecular markers, and more detailed morphological analyses will be essential for developing a robust phylogeny of Astereae.

## Data Availability

The original contributions presented in the study are included in the article/**Supplementary Material**. Further inquiries can be directed to the corresponding authors.
