# Supplementary material for: Systematic analysis of some Astereae (Asteraceae) species by Integrating pollen morphology and molecular evidence
Source: Front Plant Sci. 2025 Apr 8;16:1558995. doi: 10.3389/fpls.2025.1558995 (PMC12011805; doi:10.3389/fpls.2025.1558995)
Supplement: Supplementary file 1 [file DataSheet1.docx]

Supplementary Data

Pollen morphological descriptions of 21 species from 5 subtribes and 12 genera of Astereae. Pollen morphology of Astereae: pollen grains spheroidal or subprolate; almost circular in equatorial view and trilobate circular in polar view; tricolporate apertures with a granulate aperture membrane; the sexine is thicker than the nexine; the exine ornamentation is spinose (LM), echinate (SEM), most spine shapes are tapering to a sharp point, with one or more rows of perforations at the base, and the inter-spinal area is perforate.

**1. Subtribe. Asterinae**

**1.1 Genus: *Aster* L.**

**1.1.1 Investigated species: *Aster altaicus* Willd. (Tables 2-3, Fig 2. A-G)**

Pollen grains spheroidal. Almost circular in equatorial view and trilobate circular in polar view. Tricolporate apertures with a granulate aperture membrane. Polar length (P) = 25.72 ± 1.98 µm (M ± SD), equatorial width (E) = 26.97 ± 2.12 µm (M ± SD), P/E = 0.96 ± 0.07 (M ± SD), exine thickness (T) = 3.67 ± 0.36 µm (M ± SD), and pollen length (L) = 27.38 ± 0.93 µm (M ± SD), T/L = 0.13 ± 0.02 (M ± SD), Sexine/nexine (S/N) = 2.5. The exine ornamentation is spinose (LM), echinate (SEM). Under SEM, diameter of spinule base (D) = 3.14 ± 0.36 µm (M ± SD), spinule height (H) = 2.87 ± 0.26 µm (M ± SD), D/H = 1.10 ± 0.11 (M ± SD), and spinule spacing (Ss) = 4.02 ± 0.63 µm (M ± SD). Spine base expanded, tapering to a point, with 2 rows of perforations at the base, and the inter-spinal area is perforate.

**1.1.2 Investigated species: *Aster ageratoides* Turcz. (Tables 2-3, Fig 2. H-N)**

Pollen grains spheroidal. Almost circular in equatorial view and trilobate circular in polar view. Tricolporate apertures with a granulate aperture membrane. P = 31.35 ± 1.35 µm, E = 29.43 ± 1.88 µm, P/E = 1.07 ± 0.07, T = 4.62 ± 0.47 µm, L = 32.59 ± 1.51 µm, T/L = 0.14 ± 0.01, S/N = 1.5. The exine ornamentation is spinose (LM), echinate (SEM). Under SEM, D = 2.65 ± 0.17 µm, H = 2.98 ± 0.21 µm, D/H = 0.89 ± 0.05, Ss = 3.87 ± 0.40 µm. Spine tapering to a sharp, pointed tip, with zero or one row of perforations at the base, and the inter-spinal area is perforate.

**1.1.3 Investigated species: *Aster homochlamydeus* Hand.-Mazz. (Tables 2-3, Fig 2. O-U)**

Pollen grains spheroidal. Almost circular in equatorial view and trilobate circular in polar view. Tricolporate apertures with a granulate aperture membrane. P = 36.46 ± 2.96 µm, E = 34.93 ± 2.75 µm, P/E = 1.05 ± 0.08, T = 5.81 ± 0.72 µm, L = 39.13 ± 4.19 µm, T/L = 0.15 ± 0.01, S/N = 3. The exine ornamentation is spinose (LM), echinate (SEM). Under SEM, D = 2.98 ± 0.30 µm, H = 3.72 ± 0.39 µm, D/H = 0.80 ± 0.06, Ss = 4.44 ± 0.76 µm. Spine tapering to a sharp, with 1-2 rows of perforations at the base, and the inter-spinal area is perforate.

**1.1.4 Investigated species: *Aster turbinatus* S. Moore (Tables 2-3, Fig 3. A-G)**

Pollen grains spheroidal. Almost circular in equatorial view and trilobate circular in polar view. Tricolporate apertures with a granulate aperture membrane. P = 28.66 ± 2.03 µm, E = 29.88 ± 1.52 µm, P/E = 0.96 ± 0.08, T = 5.04 ± 0.55 µm, L = 32.17 ± 2.58 µm, T/L = 0.16 ± 0.01, S/N = 2.5. The exine ornamentation is spinose (LM), echinate (SEM). Under SEM, D = 2.86 ± 0.34 µm, H = 3.28 ± 0.42 µm, D/H = 0.88 ± 0.08, Ss = 3.83 ± 0.56 µm. Spine tapering to a sharp, with 1-2 rows of perforations at the base, and the inter-spinal area is perforate.

**1.1.5 Investigated species: *Aster taliangshanensis* Y. Ling (Tables 2-3, Fig 3. H-N)**

Pollen grains spheroidal. Almost circular in equatorial view and trilobate circular in polar view. Tricolporate apertures with a granulate aperture membrane. P = 35.69 ± 2.32 µm, E = 35.32 ± 1.96 µm, P/E = 1.01 ± 0.09, T = 6.36 ± 0.63 µm, L = 39.72 ± 2.24 µm, T/L = 0.16 ± 0.01, S/N = 2.5. The exine ornamentation is spinose (LM), echinate (SEM). Under SEM, D = 3.48 ± 0.39 µm, H = 4.14 ± 0.44 µm, D/H = 0.84 ± 0.07, Ss = 5.16 ± 0.66 µm. Spine tapering to a sharp, pointed tip, with 2 rows of perforations at the base, and the inter-spinal area is perforate.

**1.1.6 Investigated species: *Aster brachytrichus* Franch**. **(Tables 2-3, Fig 3. O-U)**

Pollen grains spheroidal. Almost circular in equatorial view and trilobate circular in polar view. Tricolporate apertures with a granulate aperture membrane. P = 27.17 ± 1.65 µm, E = 28.01 ± 1.73 µm, P/E = 0.97 ± 0.07, T = 4.45 ± 0.39 µm, L = 29.43 ± 1.61 µm, T/L = 0.15 ± 0.01, S/N = 2. The exine ornamentation is spinose (LM), echinate (SEM). Under SEM, D = 3.14 ± 0.24 µm, H = 3.11 ± 0.37 µm, D/H = 1.02 ± 0.10, Ss = 4.20 ± 0.83 µm. Spine tapering to a sharp, with 0-2 rows of perforations at the base, and the inter-spinal area is perforate.

**1.1.7 Investigated species: *Aster yunnanensis* Franch. (Tables 2-3, Fig 4. A-G)**

Pollen grains spheroidal. Almost circular in equatorial view and trilobate circular in polar view. Tricolporate apertures with a granulate aperture membrane. P = 26.19 ± 1.04 µm, E = 28.24 ± 1.58 µm, P/E = 0.93 ± 0.06, T = 4.64 ± 0.34 µm, L = 28.22 ± 1.28 µm, T/L = 0.16 ± 0.01, S/N = 2. The exine ornamentation is spinose (LM), echinate (SEM). Under SEM, D = 3.08 ± 0.36 µm, H = 3.28 ± 0.25 µm, D/H = 0.94 ± 0.09, Ss = 4.15 ± 0.61 µm. Spine tapering to a sharp, with 2 rows of perforations at the base, and the inter-spinal area is perforate.

**1.2 Genus: *Turczaninovia* DC.**

**1.2.1 Investigated species: *Turczaninovia fastigiata* (Fisch.) DC. (Tables 2-3, Fig 4. H-N)**

Pollen grains spheroidal. Almost circular in equatorial view and trilobate circular in polar view. Tricolporate apertures with a granulate aperture membrane. P = 26.04 ± 1.12 µm, E = 28.25 ± 1.28 µm, P/E = 0.92 ± 0.02, T = 4.99 ± 0.51 µm, L = 28.34 ± 1.05 µm, T/L = 0.18 ± 0.01, S/N = 2.5. The exine ornamentation is spinose (LM), echinate (SEM). Under SEM, D = 3.11 ± 0.27 µm, H = 3.41 ± 0.38 µm, D/H = 0.92 ± 0.12, Ss = 4.24 ± 0.45 µm. Spine tapering to a sharp, pointed tip, with 2 rows of perforations at the base, and the inter-spinal area is perforate.

**1.3 Genus: *Arctogeron* DC.**

**1.3.1 Investigated species: *Arctogeron gramineum* (L.) DC. (Tables 2-3, Fig 4. O-U)**

Pollen grains subprolate. Almost circular in equatorial view and trilobate circular in polar view. Tricolporate apertures with a granulate aperture membrane. P = 30.11 ± 1.50 µm, E = 26.93 ± 1.27 µm, P/E = 1.12 ± 0.05, T = 5.00 ± 0.53 µm, L = 30.73 ± 1.54 µm, T/L = 0.16 ± 0.02, S/N = 2. The exine ornamentation is spinose (LM), echinate (SEM). Under SEM, D = 2.83 ± 0.25 µm, H = 3.41 ± 0.27 µm, D/H = 0.83 ± 0.07, Ss = 3.96 ± 0.66 µm. Spine tapering to a sharp, pointed tip, with 1-2 rows of perforations at the base, and the inter-spinal area is perforate.

**1.4 Genus: *Callistephus* Cass.**

**1.4.1 Investigated species: *Callistephus chinensis* (L.) Nees (Tables 2-3, Fig 5. A-G)**

Pollen grains spheroidal. Almost circular in equatorial view and trilobate circular in polar view. Tricolporate apertures with a granulate aperture membrane. P = 33.94 ± 1.82 µm, E = 32.45 ± 1.39 µm, P/E = 1.05 ± 0.06, T = 4.57 ± 0.41 µm, L = 34.74 ± 1.45 µm, T/L = 0.13 ± 0.01, S/N = 1.5. The exine ornamentation is spinose (LM), echinate (SEM). Under SEM, D = 3.09 ± 0.33 µm, H = 3.68 ± 0.52 µm, D/H = 0.85 ± 0.08, Ss = 4.43 ± 0.72 µm. Spine base expanded, tapering to a sharp tip, with 2-3 rows of perforations at the base, and the inter-spinal area is perforate.

**1.5 Genus: *Galatella* Cass.**

**1.5.1 Investigated species: *Galatella angustissima* (Tausch) Novopokr. (Tables 2-3, Fig 5. H-N)**

Pollen grains spheroidal. Almost circular in equatorial view and trilobate circular in polar view. Tricolporate apertures with a granulate aperture membrane. P = 34.17 ± 1.25 µm, E = 31.37 ± 1.77 µm, P/E = 1.09 ± 0.05, T = 5.01 ± 0.49 µm, L = 34.71 ± 2.60 µm, T/L = 0.14 ± 0.01, S/N = 2. The exine ornamentation is spinose (LM), echinate (SEM). Under SEM, D = 3.12 ± 0.32 µm, H = 3.22 ± 0.27 µm, D/H = 0.97 ± 0.09, Ss = 4.29 ± 0.52 µm. Spine tapering to a sharp, with 2 rows of perforations at the base, and the inter-spinal area is perforate.

**2. Subtribe. Conyzinae**

**2.1 Genus: *Erigeron* L.**

**2.1.1 Investigated species: *Erigeron acris* L. (Tables 2-3, Fig 6. A-G)**

Pollen grains spheroidal. Almost circular in equatorial view and trilobate circular in polar view. Tricolporate apertures with a granulate aperture membrane. P = 23.92 ± 1.54 µm, E = 23.02 ± 1.41 µm, P/E = 1.04 ± 0.05, T = 3.72 ± 0.50 µm, L = 24.91 ± 1.83 µm, T/L = 0.15 ± 0.01, S/N = 2. The exine ornamentation is spinose (LM), echinate (SEM). Under SEM, D = 2.25 ± 0.24 µm, H = 2.58 ± 0.18 µm, D/H = 0.87 ± 0.09, Ss = 2.96 ± 0.45 µm. Spine tapering to a sharp, pointed tip, with one row of perforations at the base, and the inter-spinal area is perforate.

**2.1.2 Investigated species: *Erigeron acris* subsp. *politus* (Fr.) H. Lindb. (Tables 2-3, Fig 6. H-N)**

Pollen grains spheroidal. Almost circular in equatorial view and trilobate circular in polar view. Tricolporate apertures with a granulate aperture membrane. P = 23.59 ± 1.96 µm, E = 23.17 ± 1.37 µm, P/E = 1.02 ± 0.07, T = 3.56 ± 0.35 µm, L = 24.30 ± 1.38 µm, T/L = 0.15 ± 0.01, S/N = 2. The exine ornamentation is spinose (LM), echinate (SEM). Under SEM, D = 2.27 ± 0.18 µm, H = 2.58 ± 0.25 µm, D/H = 0.89 ± 0.10, Ss = 2.92 ± 0.25 µm. Spine tapering to a sharp, with zero or one row of perforations at the base, and the inter-spinal area is perforate.

**2.1.3 Investigated species: *Erigeron lonchophyllus* Hook. (Tables 2-3, Fig 6. O-U)**

Pollen grains spheroidal. Almost circular in equatorial view and trilobate circular in polar view. Tricolporate apertures with a granulate aperture membrane. P = 23.95 ± 2.76 µm, E = 22.69 ± 1.72 µm, P/E = 1.06 ± 0.09, T = 4.18 ± 0.38 µm, L = 26.68 ± 1.67 µm, T/L = 0.16 ± 0.01, S/N = 2. The exine ornamentation is spinose (LM), echinate (SEM). Under SEM, D = 2.70 ± 0.33 µm, H = 2.78 ± 0.30 µm, D/H = 0.97 ± 0.09, Ss = 3.53 ± 0.64 µm. Spine tapering to a sharp, pointed tip, with 2 rows of perforations at the base, and the inter-spinal area is perforate.

**2.1.4 Investigated species: *Erigeron strigosus* Muhl. ex Willd. (Tables 2-3, Fig 7. A-G)**

Pollen grains spheroidal. Almost circular in equatorial view and trilobate circular in polar view. Tricolporate apertures with a granulate aperture membrane. P = 21.80 ± 1.22 µm, E = 20.81 ± 1.08 µm, P/E = 1.05 ± 0.05, T = 3.16 ± 0.50 µm, L = 22.50 ± 1.54 µm, T/L = 0.14 ± 0.02, S/N = 2.5. The exine ornamentation is spinose (LM), echinate (SEM). Under SEM, D = 1.94 ± 0.27 µm, H = 2.08 ± 0.28 µm, D/H = 0.95 ± 0.14, Ss = 2.47 ± 0.65 µm. Spine tapering to a sharp, pointed tip, with 1-2 rows of perforations at the base, and the inter-spinal area is perforate.

**2.2 Genus: *Eschenbachia* Moench**

**2.2.1 Investigated species: *Eschenbachia japonica* (Thunb.) J. Kost. (Tables 2-3, Fig 7. H-N)**

Pollen grains spheroidal. Almost circular in equatorial view and trilobate circular in polar view. Tricolporate apertures with a granulate aperture membrane. P = 23.18 ± 1.04 µm, E = 22.19 ± 0.92 µm, P/E = 1.05 ± 0.04, T = 3.58 ± 0.35 µm, L = 25.80 ± 1.19 µm, T/L = 0.14 ± 0.01, S/N = 1.5. The exine ornamentation is spinose (LM), echinate (SEM). Under SEM, D = 2.34 ± 0.21 µm, H = 2.45 ± 0.35 µm, D/H = 0.97 ± 0.14, Ss = 3.05 ± 0.49 µm. Spine tapering to a sharp, with 1-2 rows of perforations at the base, and the inter-spinal area is perforate.

**3. Subtribe. Lagenophorinae**

**3.1 Genus: *Myriactis* Less.**

**3.1.1 Investigated species: *Myriactis wallichii* Less. (Tables 2-3, Fig 7. O-U)**

Pollen grains spheroidal. Almost circular in equatorial view and trilobate circular in polar view. Tricolporate apertures with a granulate aperture membrane. P = 32.06 ± 2.07 µm, E = 30.32 ± 1.75 µm, P/E = 1.06 ± 0.05, T = 5.50 ± 0.54 µm, L = 32.33 ± 2.16 µm, T/L = 0.17 ± 0.01, S/N = 2.5. The exine ornamentation is spinose (LM), echinate (SEM). Under SEM, D = 2.97 ± 0.35 µm, H = 3.74 ± 0.52 µm, D/H = 0.80 ± 0.07, Ss = 4.16 ± 0.62 µm. Spine tapering to a sharp, pointed tip, with one row of perforations at the base, and the inter-spinal area is perforate.

**4. Subtribe. Grangeinae**

**4.1 Genus: *Dichrocephala* L'Hér. ex DC.**

**4.1.1 Investigated species: *Dichrocephala benthamii* C. B. Clarke (Tables 2-3, Fig 8. A-G)**

Pollen grains spheroidal. Almost circular in equatorial view and trilobate circular in polar view. Tricolporate apertures with a granulate aperture membrane. P = 22.86 ± 2.52 µm, E = 21.93 ± 2.08 µm, P/E = 1.04 ± 0.08, T = 3.90 ± 0.37 µm, L = 23.90 ± 1.62 µm, T/L = 0.16 ± 0.01, S/N = 2. The exine ornamentation is spinose (LM), echinate (SEM). Under SEM, D = 2.61 ± 0.44 µm, H = 2.83 ± 0.55 µm, D/H = 0.93 ± 0.08, Ss = 3.37 ± 0.92 µm. Spine tapering to a sharp, with 1-2 rows of perforations at the base, and the inter-spinal area is perforate.

**5. Subtribe. Solidagininae**

**5.1 Genus: *Solidago* L.**

**5.1.1 Investigated species: *Solidago altissima* L. (Tables 2-3, Fig 8. H-N)**

Pollen grains spheroidal. Almost circular in equatorial view and trilobate circular in polar view. Tricolporate apertures with a granulate aperture membrane. P = 25.70 ± 2.09 µm, E = 25.38 ± 1.08 µm, P/E = 1.01 ± 0.05, T = 4.28 ± 0.32 µm, L = 26.76 ± 1.24 µm, T/L = 0.16 ± 0.01, S/N = 2.5. The exine ornamentation is spinose (LM), echinate (SEM). Under SEM, D = 2.67 ± 0.23 µm, H = 2.88 ± 0.18 µm, D/H = 0.93 ± 0.09, Ss = 3.67 ± 0.69 µm. Spine tapering to a sharp, with 1-2 rows of perforations at the base, and the inter-spinal area is perforate.

**6. Subtribe. Uncertain**

**6.1 Genus: *Formania* W. W. Sm. & J. Small**

**6.1.1 Investigated species: *Formania mekongensis* W. W. Sm. & J. Small (Tables 2-3, Fig 5. O-U)**

Pollen grains spheroidal. Almost circular in equatorial view and trilobate circular in polar view. Tricolporate apertures with a granulate aperture membrane. P = 26.05 ± 1.43 µm, E = 25.26 ± 2.30 µm, P/E = 1.04 ± 0.11, T = 4.64 ± 0.44 µm, L = 28.22 ± 1.73 µm, T/L = 0.16 ± 0.01, S/N = 2.5. The exine ornamentation is spinose (LM), echinate (SEM). Under SEM, D = 2.83 ± 0.26 µm, H = 2.78 ± 0.23 µm, D/H = 1.02 ± 0.09, Ss = 4.05 ± 0.41 µm. Spine tapering to a sharp, with one row of perforations at the base, and the inter-spinal area is perforate.

**6.2 Genus: *Nannoglottis* Maxim.**

**6.2.1 Investigated species: *Nannoglottis carpesioides* Maxim. (Tables 2-3, Fig 8. O-U)**

Pollen grains spheroidal. Almost circular in equatorial view and trilobate circular in polar view. Tricolporate apertures with a granulate aperture membrane. P = 32.12 ± 2.04 µm, E = 30.07 ± 1.93 µm, P/E = 1.07 ± 0.07, T = 5.51 ± 0.46 µm, L = 33.33 ± 1.23 µm, T/L = 0.17 ± 0.01, S/N = 2. The exine ornamentation is spinose (LM), echinate (SEM). Under SEM, D = 3.03 ± 0.31 µm, H = 3.66 ± 0.34 µm, D/H = 0.83 ± 0.06, Ss = 4.61 ± 0.74 µm. Spine tapering to a sharp, with zero or one row of perforations at the base, and the inter-spinal area is perforate.
